# Resveratrol-Enhanced Human Neural Stem Cell-Derived Exosomes Mitigate MPP+-Induced Neurotoxicity Through Activation of AMPK and Nrf2 Pathways and Inhibition of the NLRP3 Inflammasome in SH-SY5Y Cells

**DOI:** 10.3390/life15020294

**Published:** 2025-02-13

**Authors:** Ming-Chang Chiang, Yu-Ping Yang, Christopher J. B. Nicol, Tairui Chiang, Chiahui Yen

**Affiliations:** 1 Department of Life Science, College of Science and Engineering, Fu Jen Catholic University, New Taipei City 242, Taiwan; 2 Sylvester Comprehensive Cancer Center, Miller School of Medicine, University of Miami, Miami, FL 33136, USA; yyang22@med.miami.edu; 3 Department of Biochemistry and Molecular Biology, Miller School of Medicine, University of Miami, Miami, FL 33136, USA; 4 Departments of Pathology & Molecular Medicine and Biomedical & Molecular Sciences, and Cancer Biology and Genetics Division, Sinclair Cancer Research Institute, Queen’s University, Kingston, ON K7L 3N6, Canada; nicolc@queensu.ca; 5 Ames Middle School, Ames, IA 50014, USA; 6 New Taipei Municipal Jinhe High School, New Taipei City 235, Taiwan; 7 Department of International Business, Ming Chuan University, Taipei 111, Taiwan

**Keywords:** resveratrol, AMPK, exosomes, MPP^+^, neuroprotection

## Abstract

Parkinson’s disease (PD) is a progressive neurodegenerative disorder primarily characterized by the loss of dopaminergic neurons in the substantia nigra. Mitochondrial dysfunction, oxidative stress, and neuroinflammation are recognized as critical pathological mechanisms driving neurodegeneration in PD. Exosome (Exo)-based therapies, particularly those derived from human neural stem cells (hNSCs), offer promising neuroprotective effects due to their ability to transfer bioactive molecules that modulate cellular processes. Resveratrol (RES), a polyphenolic compound with potent antioxidant and anti-inflammatory properties, has been shown to enhance the therapeutic potential of stem cell (SC)-derived Exos. This study investigated the neuroprotective effects of RES-treated hNSCs-derived Exos (RES-hNSCs-Exos) on SH-SY5Y cells exposed to 1-methyl-4-phenylpyridinium (MPP^+^), a neurotoxin commonly used to model Parkinsonian neurotoxicity. Treating SH-SY5Y cells with MPP^+^ led to significant reductions in cell viability, mitochondrial dysfunction, increased oxidative stress, and the activation of inflammatory pathways. Treatment with RES-hNSCs-Exos rescued SH-SY5Y cells from MPP^+^-induced toxicity by improving cell viability, enhancing ATP production, increasing mitochondrial biogenesis, and reducing reactive oxygen species (ROS) generation. The findings also demonstrated the increased expression of essential genes involved in mitochondrial biogenesis, such as PGC1α, NRF1, and Tfam, indicating improved mitochondrial function in the presence of RES-hNSCs-Exos. Further analysis revealed that these protective effects were mediated by activating the AMP-activated protein kinase (AMPK) and Nrf2 signaling pathways, which promoted mitochondrial health and reduced oxidative stress. Moreover, RES-hNSCs-Exos treatment suppressed neuroinflammation by downregulating NLRP3 inflammasome activation and reducing the secretion of pro-inflammatory cytokines IL-1β and IL-18. In conclusion, the results suggest that RES-hNSCs-Exos exhibit potent neuroprotective effects against MPP^+^-induced neurotoxicity by enhancing mitochondrial function, reducing oxidative stress, and inhibiting neuroinflammation. These findings highlight the potential of hNSCs-Exos as a novel therapeutic strategy for neurodegenerative diseases like PD, with RES as a valuable enhancer of Exos efficacy.

## 1. Introduction

PD is a progressive neurodegenerative disorder characterized by the loss of dopaminergic neurons in the substantia nigra, leading to motor dysfunction, cognitive impairment, and other debilitating symptoms [1,2,3,4,5]. The pathogenesis of PD is multifactorial, involving oxidative stress, mitochondrial dysfunction, neuroinflammation, and impaired proteostasis [6,7,8,9]. Despite the availability of symptomatic treatments, such as dopamine replacement therapies, there are no disease-modifying treatments that can halt or reverse neurodegeneration. Thus, the need for innovative therapeutic strategies that target multiple pathways involved in PD progression is critical. Mitochondrial dysfunction and oxidative stress are key contributors to the neurodegeneration seen in PD [10]. Impairment of mitochondrial biogenesis, disruption of the mitochondrial membrane potential, and excessive ROS production are all PD hallmarks [11]. Moreover, neuroinflammation, mediated by the activation of the NLRP3 inflammasome and the release of pro-inflammatory cytokines, is pivotal in exacerbating neuronal damage [12]. Strategies that can restore mitochondrial function, reduce oxidative stress, and suppress neuroinflammation are considered promising approaches for neuroprotection.

Exos-based therapies have emerged as a novel and promising approach to neurodegenerative diseases [13,14]. Exos are small extracellular vesicles secreted by cells, including neural stem cells (NSCs), and they carry a diverse array of bioactive molecules, such as proteins, lipids, and RNA, that can modulate cellular processes in recipient cells [15,16]. Recent studies have demonstrated that Exos derived from hNSCs can promote neuronal survival, enhance mitochondrial function, and reduce inflammation in various models of neurodegeneration [15,17]. Additionally, resveratrol, a natural polyphenolic compound, possesses well-documented antioxidant and anti-inflammatory properties [18,19,20]. These properties help combat cellular damage caused by oxidative stress and inflammation. Resveratrol enhances cellular defense mechanisms by activating signaling pathways like SIRT1 (Sirtuin 1), promoting mitochondrial function, and reducing apoptosis [21]. Notably, the compounds that activate AMPK-dependent pathways have drawn attention as potential therapeutics against PD [22]. AMPK is an enzyme that plays a crucial role in cellular energy homeostasis [23]. Th activation of AMPK can have various protective effects on cells, including those in the brain [22,24]. In studies using SH-SY5Y cells, a commonly used human neuroblastoma cell line, resveratrol or other has been shown to mediate AMPK-dependent pathways [25,26]. This activation can lead to increased cellular antioxidant defenses, enhanced mitochondrial function, and reduced inflammation, all of which are important for protecting neurons against neurotoxicity.

This study aimed to investigate the neuroprotective effects of RES-hNSCs-Exos in an in vitro model of PD. The neurotoxin MPP^+^, a metabolite of 1-methyl-4-phenyl-1,2,3,6-tetrahydropyridine (MPTP), is widely used to model PD in vitro as it induces neurotoxicity by targeting dopaminergic neurons, causing mitochondrial dysfunction, oxidative stress, and apoptosis [27,28,29]. It is hypothesized that hNSCs-Exos would mitigate MPP+-induced neurotoxicity by enhancing mitochondrial function, reducing oxidative stress, and inhibiting neuroinflammation via the AMPK and Nrf2 signaling pathways. By elucidating the mechanisms through which hNSCs-Exos confer neuroprotection, this study contributes to the growing body of evidence supporting the therapeutic potential of Exos-based strategies for neurodegenerative diseases. Furthermore, using resveratrol to augment the efficacy of hNSCs-Exos offers a promising avenue for developing more potent neuroprotective therapies.

## 2. Materials and Methods

### 2.1. Cell Culture

Human neuroblastoma SH-SY5Y cells were obtained from ATCC and maintained in Dulbecco’s Modified Eagle Medium (DMEM) supplemented with 10% fetal bovine serum (FBS), 1% penicillin-streptomycin, and 1% L-glutamine. Cells were incubated at 37 °C in a humidified atmosphere of 5% CO_2_. hNSCs were cultured in NSC medium containing Neurobasal medium, B27 supplement, bFGF (20 ng/mL), and EGF (20 ng/mL). Cells were subcultured every 4–5 days. SH-SY5Y cells are a widely used dopaminergic neuronal cell line for studying PD [30].

### 2.2. Preparation of hNSCs-Exos

hNSCs were cultured using a neural stem cell serum-free medium (NSC SFM; Gibco, Thermo Fisher Scientific, Waltham, MA, USA following standard cell culture protocols. To investigate the effects of the activator (resveratrol) and the antagonist (Compound C) of AMPK, hNSCs were treated with either 10 µM Resveratrol (Sigma-Aldrich, St. Louis, MO, USA) or 10 µM Compound C (Sigma-Aldrich) for 48 h. The Total Exos Isolation Reagent (Thermo Fisher Scientific) was utilized for Exo isolation. This reagent allows the rapid and efficient enrichment of intact Exos from the cell culture medium. The isolation mechanism involves binding water molecules and forcing vesicles, such as Exos, out of the solution. Following this, a short, low-speed centrifugation was performed. The Exos isolation procedure involved adding the reagent to the cell culture medium, which was incubated overnight at 4 °C. hNSCs-derived Exos were isolated by ultracentrifugation. Briefly, conditioned media from hNSC cultures were collected and subjected to sequential centrifugation at 300× *g* for 10 min, 2000× *g* for 30 min, and 10,000× *g* for 1 h to remove cell debris. The supernatant was then ultracentrifuged at 100,000× *g* for 70 min to pellet the Exos. After centrifugation, the Exo pellet was resuspended in PBS or an appropriate buffer and quantified by a BCA protein assay.

### 2.3. Cell Treatment Groups

hNSCs-Exos were then aliquoted and stored at −20 °C until use. The SH-SY5Y cells were then divided into four treatment groups: a. Control (CON) group: SH-SY5Y cells cultured in DMEM medium without treatment for 72 h. b. MPP^+^ group: SH-SY5Y cells treated with 50 μM MPP^+^ in DMEM medium for 24 h. After 24 h, the MPP^+^-containing medium was replaced with a fresh medium. Cells were cultured in the medium for an additional 48 h. c. Resveratrol (RES) group: SH-SY5Y cells were treated with 50 μM MPP^+^ in DMEM medium for 24 h. After 24 h, the MPP^+^-containing medium was replaced with a medium containing 10 μg/mL RES (10 μM)-treated hNSCs-Exos. Cells were cultured in this medium for an additional 48 h. d. Compound C (CC) group: SH-SY5Y cells were treated with 50 μM MPP^+^ in DMEM medium for 24 h. After 24 h, the MPP^+^-containing medium was replaced with medium containing 10 μg/mL CC (10 μM)-treated hNSCs-Exos. Cells were cultured in this medium for an additional 48 h.

### 2.4. Cell Viability Assay

Cell viability was assessed using the MTT assay. SH-SY5Y cells were seeded in the 35 mm dish and treated as described [31]. After treatment, 20 µL of MTT reagent (5 mg/mL) was added to each well, and the cells were incubated for 1 h at 37 °C. The formazan crystals formed were dissolved in DMSO, and absorbance was measured at 570 nm using a microplate reader.

### 2.5. Caspase-3 and Caspase-9 Activity Assay

Caspase-3 and caspase-9 activities were measured using fluorometric protease assays. SH-SY5Y cell lysates were incubated with the DEVD-AFC substrate for caspase-3 and the LEHD-FMK substrate for caspase-9. Fluorescence was measured at an excitation/emission wavelength of 400/505 nm for caspase-3 and 485/535 nm for caspase-9, and the results were normalized to the protein concentration.

### 2.6. LIVE/DEAD Cell Viability Assays

SH-SY5Y cells were cultured in standard growth medium (DMEM/F12 supplemented with 10% fetal bovine serum and 1% penicillin-streptomycin) and maintained in a humidified atmosphere with 5% CO_2_ at 37 °C. Cells were seeded in 24-well plates at a density of approximately 1 × 10⁵ cells/well. After 24 h, the cells were treated with MPP^+^ to induce neurotoxicity, followed by RES-hNSCs-Exos after the designated treatment period. The cells were then incubated with a mixture of calcein-AM (2 µM) and ethidium homodimer-1 (4 µM) diluted in PBS for 30 min at 37 °C. A two-color fluorescence-based LIVE/DEAD cell viability assay was performed to distinguish live and dead cells, using calcein-AM and ethidium homodimer-1 staining.

### 2.7. Quantitative PCR (qPCR)

Total RNA was extracted from SH-SY5Y cells using TRIzol reagent, and cDNA was synthesized using a reverse transcription kit. qPCR was performed using SYBR Green Master Mix to quantify the expression of target genes (AMPK, Bcl-2, CREB, PGC-1α, NRF1, Tfam, Nrf2, NLRP3, ASC, and caspase-1) relative to the housekeeping gene GAPDH. The sequences of primers were as follows: AMPK (5′-GCTGTGGATCGCCAAATTAT-3′ and 5′-CACGTGCTCATCATCGAAAG-3′); Bcl-2 (5′-GGCTGGGATGCCTTTGTG-3′ and 5′-CAGCCAGGAGAAATCAAACAGA-3′); CREB (5′-CCAAGCTTATGGATCCTCCTGGAGAGAAGATGG-3′ and 5′-GCCTCGAGAA-GCACATTGACGCTCCTGAC-3′); PGC1α (5′-TGAGAGGGCCAAGCAAAG-3′ and 5′-ATAAATCACACGGCGCTCTT-3′); NRF-1 (5′-CCATCTGGTGGCCTGAAG-3′ and 5′-GTGCCTGGGTCCATGAAA-3′); Tfam (5′-GAACAACTACCCATATTTAAAGCTCA-3′ and 5′-GAATCAGGAAGTTCCCTCCA-3′); Nrf2 (5′-ACACGGTCCACAGCTCATC-3′ and 5′-TGTC- AATCAAATCCATGTCCTG-3′); NLRP3 (5′-TGCCCGTCTGGGTGAGA-3′ and 5′-CCGG-TGCTCCTTGATGAGA-3′); ASC (5′-CGCGAGGGTCACAAACGT-3′ and 5′-TGCTC- ATCCGTCAGGACCTT-3′); caspase-1 (5′-AATTTTCCGCAAGGTTCGATT-3′ and 5′-ACTCTTTCAGTGGTGGGCATCT-3′); and GADPH (5′-CATCTTCTTTTGCGTCGCCA-3′ and 5′-TTAAAAGCAGCCCTGGTGACC-3′). Gene expression levels were calculated using the 2^−ΔΔCt^ method.

### 2.8. Measurement of ATP Levels

ATP levels in SH-SY5Y cells were determined using a luminescence-based ATP assay. Cell lysates were prepared, and ATP content was measured using a commercial ATP detection kit according to the manufacturer’s instructions. Luminescence was recorded using a microplate reader, and the results were normalized to the protein content.

### 2.9. Mitochondrial Mass Analysis

Mitochondrial mass was analyzed using Mitotracker Green™ FM, a fluorescent dye that labels active mitochondria. SH-SY5Y cells were stained with Mitotracker Green™ (200 nM) for 30 min at 37 °C. Cells were washed and imaged using a fluorescence microscope. Quantification of fluorescence intensity was performed using ImageJ software (Version 1.53t).

### 2.10. ROS Assay

ROS levels were measured using the DCFH-DA assay. SH-SY5Y cells were incubated with 10 µM DCFH-DA for 30 min at 37 °C in the dark. Fluorescence intensity, indicative of intracellular ROS levels, was measured using a microplate reader at an excitation/emission wavelength of 485/535 nm. DHE staining was also used to visualize cell ROS production, with images captured using fluorescence microscopy.

### 2.11. Nrf2 Protein Activity Assay

The activity of the nuclear factor erythroid 2-related factor 2 (Nrf2) in the nucleus was measured using the Nrf2 transcription factor assay kit (Invitrogen), which quantifies the active, DNA-binding form of Nrf2 to assess its transcriptional activity. Following the treatments, cells were harvested, and nuclear proteins were extracted using a nuclear extraction kit (Abcam, Cambridge, UK). Briefly, cells were ly, sed in a hypotonic buffer and then centrifuged at 12,000× *g* for 10 min to separate the cytoplasmic fraction. The nuclear pellet was resuspended in an atomic extraction buffer containing protease and phosphatase inhibitors and centrifuged again to collect the atomic proteins’ supernatant. The nuclear extracts were then subjected to the Nrf2 transcription factor assay kit per the manufacturer’s protocol. This ELISA-based assay utilizes a specific DNA sequence that binds active Nrf2 in the nuclear fraction; Nrf2 activity was expressed as absorbance at 450 nm.

### 2.12. Immunostaining

SH-SY5Y cells were fixed in 4% paraformaldehyde, permeabilized with 0.1% Triton X-100, and blocked with 5% BSA. Cells were incubated with primary antibodies against caspase 3, Nrf2, and NLRP3, followed by Alexa Fluor^®^-conjugated secondary antibodies. Nuclei were counterstained with DAPI. Fluorescence images were captured using a confocal microscope, and the fluorescence intensity was quantified using ImageJ software.

### 2.13. Caspase-1 Activity Assay

Caspase-1 activity was assessed using the Caspase-Glo^®^ 1 inflammasome assay. SH-SY5Y cells were lysed, and the lysates were incubated with the substrate for caspase-1. Luminescence was measured using a microplate reader, and the results were normalized to the protein concentration.

### 2.14. ELISA for Cytokine Detection

The secretion levels of IL-1β and IL-18 in the culture supernatants were measured using ELISA kits. Supernatants were collected after treatment, and cytokine levels were quantified according to the manufacturer’s protocol. Absorbance was measured at 450 nm using a microplate reader.

### 2.15. Statistical Analysis

Data are presented as mean ± SEM from at least three independent experiments. Statistical analysis was performed using a one-way ANOVA followed by a Tukey’s post hoc test for multiple comparisons. A *p*-value < 0.001 was considered statistically significant.

## 3. Results

### 3.1. hNSCs-Exos Alleviate MPP^+^-Induced Cell Death, Caspase Activation, and Toxicity

**Figure 1:**
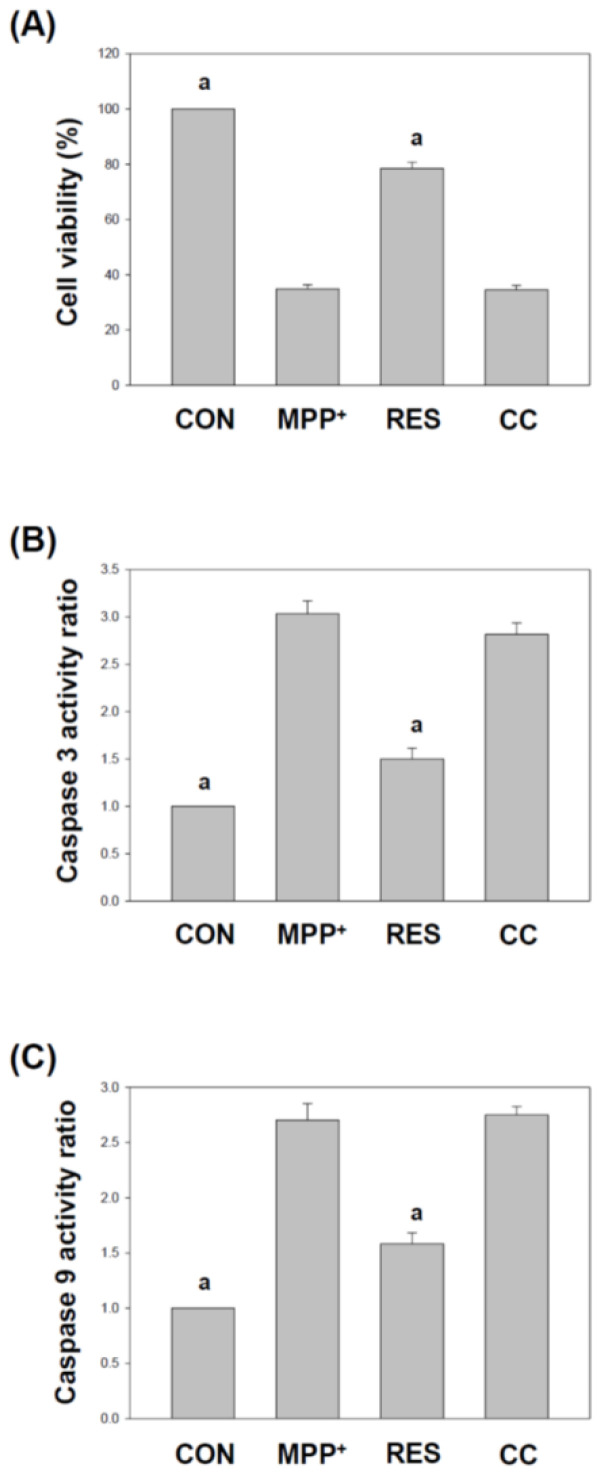
**hNSCs-Exos** Rescue SH-SY5Y Cell Viability from MPP^+^-Induced Toxicity. The experiment was divided into four groups, each representing a different treatment condition: Control (CON) group: SH-SY5Y cells cultured in DMEM medium without treatment for 72 h. b. MPP^+^ group: SH-SY5Y cells were treated with 50 μM MPP^+^ for 24 h, followed by culture medium for 48 h. Resveratrol (RES) group: SH-SY5Y cells were treated with 50 μM MPP^+^ for 24 h, followed by treatment with 10 μg/mL RES-hNSCs-Exos for another 48 h. Compound C (CC) group: SH-SY5Y cells were treated with 50 μM MPP^+^ for 24 h, followed by treatment with 10 μg/mL CC-hNSCs-Exos for another 48 h. The figure presents the results of two measurements: (**A**) Cell viability, assessed using the MTT assay, determining the amount of cellular protein to indicate cell viability. (**B**,**C**) Caspase-3 and Caspase-9 activity: Caspase activities were measured using a fluorometric protease assay. Caspase-3 activity was assessed using the DEVD-AFC substrate, and caspase-9 activity was measured using the LEHD-FMK substrate. The data were normalized to percentages relative to the CON group and are presented as mean ± SEM from three independent experiments. The designation “a” indicates a significant difference compared to SH-SY5Y cells treated with MPP^+^ alone (*p* < 0.001, one-way ANOVA).

- SH-SY5Y cells are commonly utilized in PD research due to their human origin and neuronal characteristics [30]. While differentiation into a dopaminergic phenotype is often used to better model PD pathology, our previous experiments led us to focus on undifferentiated SH-SY5Y cells [26,31]. Our data show that the undifferentiated state offers a more sensitive and consistent model for assessing the effects of drugs, particularly in terms of mitochondrial function, oxidative stress, and inflammatory responses. Moreover, undifferentiated SH-SY5Y cells maintain their ability to proliferate, facilitating better evaluation of cell viability and neuroprotection under experimental conditions. To explore the neuroprotective effects of RES-hNSCs-Exos on SH-SY5Y cells, we utilized a commonly used in vitro model for studying neurodegenerative diseases. SH-SY5Y cells were exposed to MPP^+^, a neurotoxin that induces oxidative stress and mitochondrial dysfunction, mimicking the cellular damage observed in PD [29,32]. Figure 1 investigates the ability of hNSCs-Exos to rescue SH-SY5Y cell viability following MPP^+^-induced neurotoxicity. The MTT assay (1A) was used to assess cell viability under different treatment conditions, while the caspase-3 and caspase-9 assays (1B and 1C) evaluated apoptosis. MPP^+^ group: MPP^+^ exposure significantly reduced cell viability to approximately 40% compared to the Control (CON) group (*p* < 0.001), indicating high toxicity and neurodegenerative effects on SH-SY5Y cells. Resveratrol (RES) group: treatment with RES-hNSCs-Exos restored cell viability to about 80%, suggesting a solid neuroprotective effect of RES-enhanced Exos against MPP^+^-induced toxicity. Compound C (CC) group: in this group, Exos from hNSCs treated with Compound C (an AMPK inhibitor) restored cell viability to around 40%, significantly less than the RES group, implying that AMPK activation has a role in the neuroprotection provided by hNSC-Exos. Immunofluorescence staining was used to visualize the levels of caspase 3 protein in the treated SH-SY5Y cells (Figure S1). The cells exposed to MPP^+^ displayed increased green fluorescence, indicating high caspase 3 expression. Notably, the cells treated with RES-hNSCs-Exos showed a significant decrease in caspase 3 levels compared to those treated with MPP^+^ alone. In contrast, the CC-hNSCs-Exos did not show any reduction in caspase 3 expression compared to the MPP^+^ group. The results highlight the protective effects of RES-hNSCs-Exos in reducing MPP^+^-induced cytotoxicity and apoptosis. Compound C-treated hNSCs-derived Exos (CC-hNSCs-Exos) did not exhibit neuroprotective effects, indicating that AMPK plays a crucial role in the neuroprotective mechanism of hNSC-Exos.

**Figure 2:**
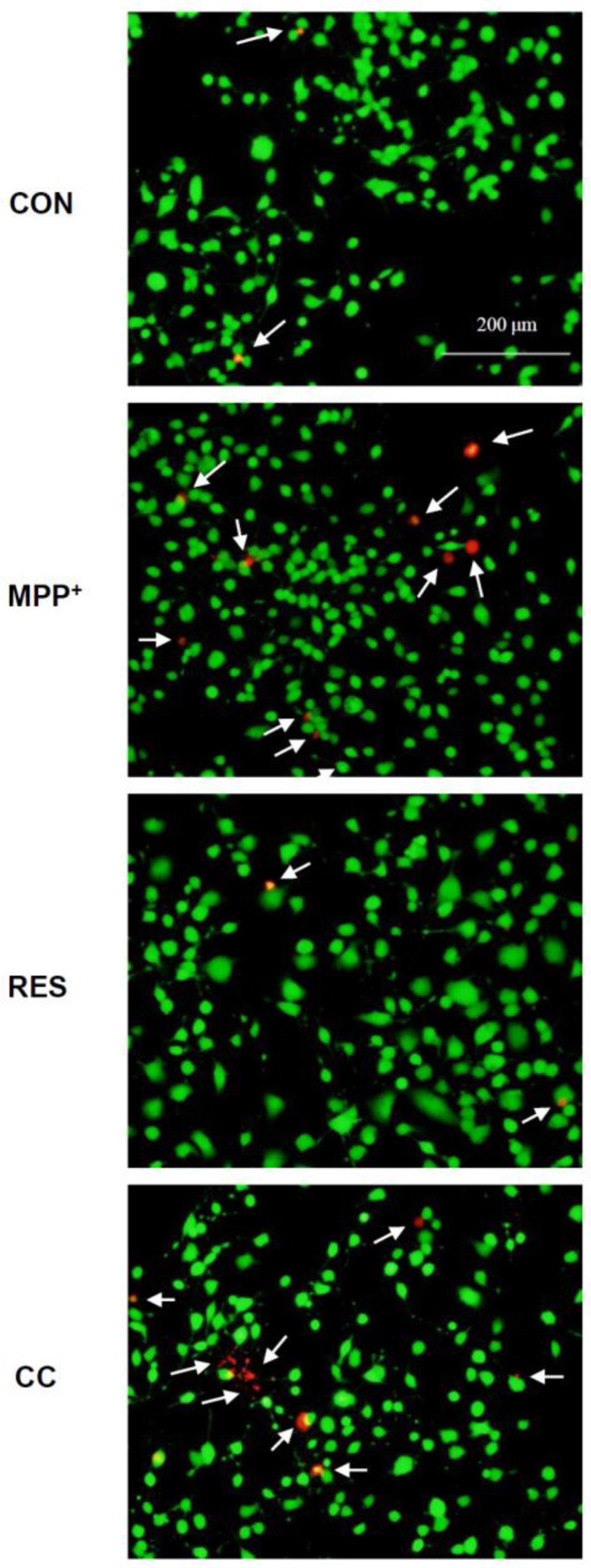
**hNSCs-Exos Prevent** MPP^+^**-Induced Decrease in SH-SY5Y Cell Viability.** Cells were treated according to the experimental setup described in Figure 1. After treatment, SH-SY5Y cells were collected and subjected to a two-color assay using fluorescent dyes to distinguish live and dead cells. Microphotographs were captured to represent the cell populations stained with calcein-AM (green, indicating live cells) and ethidium homodimer-1 (red, indicating dead cells). White arrows indicate the presence of dead cells. A representative image from three independent experiments is presented, with a scale bar indicating 200 μm.

Figure 2 further supports these findings by using live/dead cell staining to visualize the population of viable and dead cells after treatment. The calcein-AM and ethidium homodimer-1 staining allowed for a visual distinction between live (green) and dead (red) cells, illustrating the protective role of hNSCs-Exos in preventing MPP^+^-induced cell death. Together, these figures establish the foundation for understanding how RES-hNSCs-Exos confer neuroprotection by rescuing cell viability and reducing apoptosis in a neurotoxic environment.

### 3.2. hNSCs-Exos Increase AMPK, Bcl-2, and CREB Gene Expression Levels in SH-SY5Y Cells Exposed to MPP^+^

**Figure 3:**
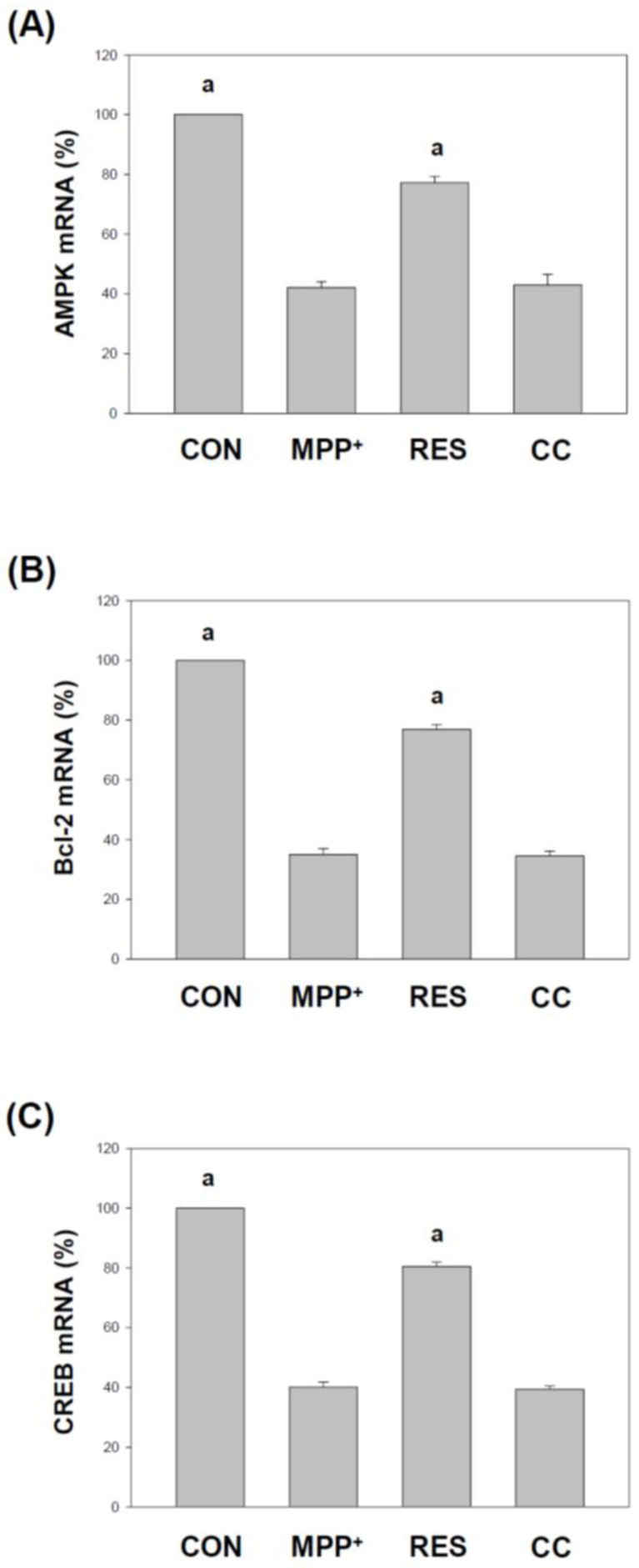
**hNSCs-Exos Increase AMPK, Bcl-2, and CREB Gene Expression Levels** in SH-SY5Y Cells Exposed to MPP^+^. Cells were treated following the protocol described in Figure 1. The expression levels of AMPK (**A**), Bcl-2 (**B**), and CREB (**C**) transcripts in the specified cells were analyzed using the qPCR technique. RNA from the selected cells was extracted and reverse transcribed into cDNA. A qPCR analysis of the target genes was performed, and the GAPDH reference gene was normalized. Each reaction was conducted in triplicate for accuracy and consistency across three independent experiments. The AMPK, Bcl-2, and CREB transcripts (**A**–**C**) are presented as percentages relative to the CON and represented as mean ± SEM values from the three independent experiments. The designation “a” indicates a significant difference compared to SH-SY5Y cells treated with MPP^+^ alone (*p* < 0.001, one-way ANOVA).

By assessing the expression levels of these genes via qPCR, Figure 3 demonstrates how RES-hNSCs-Exos activate crucial signaling pathways, such as energy regulation (AMPK) and apoptosis inhibition (Bcl-2), and enhance neuroprotective gene transcription (CREB), thus supporting the survival of SH-SY5Y cells in the face of MPP^+^-induced neurotoxicity. MPP^+^ group: Treatment with MPP^+^ significantly reduced AMPK, Bcl-2, and CREB gene expression compared to the control group (*p* < 0.001), indicating that MPP^+^ inhibits AMPK, Bcl-2, and CREB activation, which may contribute to the observed neurotoxicity. RES group: SH-SY5Y cells treated with RES-hNSCs-Exos exhibited a significant increase in AMPK, Bcl-2, and CREB gene expression compared to the MPP^+^ group (*p* < 0.001). This suggests that RES-enhanced Exos can upregulate AMPK expression, which may contribute to their neuroprotective effects. CC group: CC-hNSCs-Exos showed no increase in AMPK, Bcl-2, and CREB gene expression compared with the MPP^+^ group, further supporting the protective role of AMPK in mediating RES-treated Exos.

### 3.3. hNSCs-Exos Improve Cellular Cellular Energy and Mitochondrial Health in MPP^+^-Treated Cells

**Figure 4:**
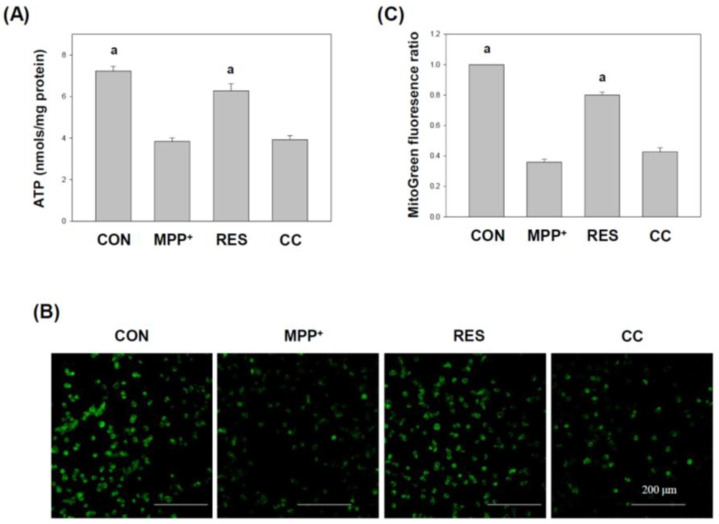
**hNSCs-Exos Increase ATP Levels and Mitochondrial Mass in SH-SY5Y Cells Exposed to** MPP^+^**.** Cells were treated as outlined in Figure 1 to examine the effects of RES-hNSCs-Exos on cellular energy levels and mitochondrial mass in SH-SY5Y cells under MPP^+^-induced neurotoxic stress. (**A**) Cellular ATP levels were measured using an ATP assay from the lysates of treated SH-SY5Y cells. The data show that RES-hNSCs-Exos significantly increased ATP production, indicating enhanced cellular energy metabolism in cells exposed to MPP^+^ (*p* < 0.001). (**B**) Mitochondrial mass was assessed in SH-SY5Y cells using Mitotracker Green™ dye, which fluoresces in green. Representative images of mitochondrial mass are displayed with a scale bar of 200 μm, visually showing that RES-hNSCs-Exos increased mitochondrial mass compared to MPP^+^-treated cells. (**C**) Mitochondrial mass was quantified and normalized to cell number to ensure accuracy. Results show that cells treated with RES-hNSCs-Exos significantly increased mitochondrial mass compared to MPP^+^-only treated cells (*p* < 0.001). The designation “a” indicates a significant difference compared to SH-SY5Y cells treated with MPP^+^ alone (*p* < 0.001, one-way ANOVA).

Figure 4 reveals the effects of RES-treated hNSCs-Exos on mitochondrial function in SH-SY5Y cells exposed to MPP^+^-induced neurotoxicity. Mitochondrial dysfunction is a hallmark of neurodegenerative diseases, and restoring mitochondrial health is crucial for neuroprotection [33,34]. Figure 4 assesses two critical aspects of mitochondrial function: ATP levels (Panel A): MPP^+^-treated cells typically experience a decrease in ATP production, indicating impaired mitochondrial energy metabolism. Treatment with RES-hNSCs-Exos significantly increased ATP levels, suggesting improved mitochondrial energy output and overall cellular energy balance. Mitochondrial mass (Panels B and C): Using Mitotracker Green™ staining, this study visually demonstrates that RES-hNSCs-Exos increase mitochondrial mass compared to MPP^+^-treated cells. Quantifying this staining confirms a significant rise in mitochondrial content, indicating enhanced mitochondrial biogenesis and health in response to hNSCs-Exos.

### 3.4. hNSCs-Exos Increase PGC1α, NRF1, and Tfam Gene Expression Levels in SH-SY5Y Cells Exposed to MPP^+^

**Figure 5:**
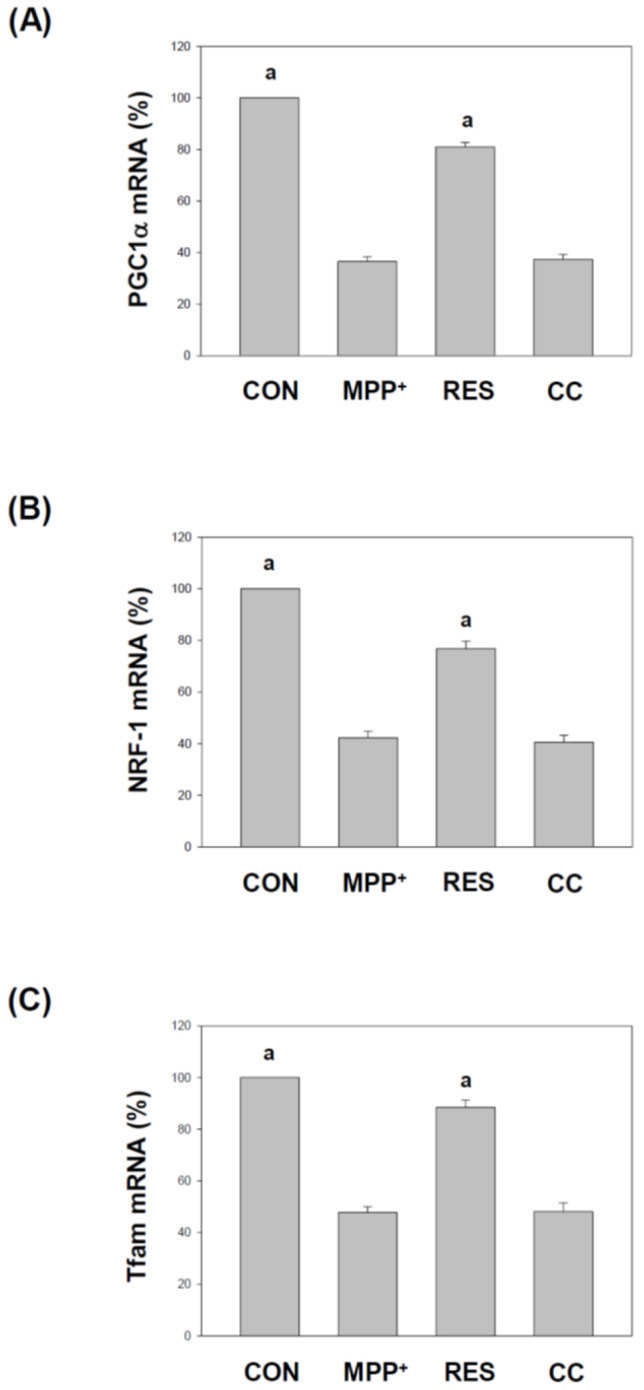
**hNSCs-Exos Increase PGC1α, NRF1, and Tfam Gene Expression Levels in SH-SY5Y Cells Exposed to** MPP^+^**.** Cells were treated following the protocol in Figure 1 to investigate the effect of RES-hNSCs-Exos on the expression of genes critical for mitochondrial biogenesis and function in MPP^+^-treated SH-SY5Y cells. (**A**) PGC1α expression: qPCR was used to assess PGC1α expression, a master regulator of mitochondrial biogenesis. Treatment with RES-hNSCs-Exos significantly increased PGC1α expression compared to cells treated with MPP^+^ alone, suggesting enhanced mitochondrial biogenesis. (**B**) NRF1 expression was measured by qPCR, indicating that RES-hNSCs-Exos upregulated this gene, which regulates mitochondrial gene expression and respiratory chain function. (**C**) The transcript levels of Tfam, essential for mitochondrial DNA replication and maintenance, were significantly increased in cells treated with RES-hNSCs-Exos compared to MPP^+^ alone, pointing to improved mitochondrial function. RNA was extracted from the cells, reverse transcribed into cDNA, and subjected to qPCR, with GAPDH as the reference gene. The data represent the mean ± SEM from three independent experiments. The designation “a” indicates a significant difference compared to SH-SY5Y cells treated with MPP^+^ alone (*p* < 0.001, one-way ANOVA).

Figure 5 explores the impact of RES-hNSCs-Exos on the expression of genes critical for mitochondrial biogenesis and function in SH-SY5Y cells subjected to MPP^+^-induced neurotoxicity. Mitochondrial biogenesis is essential for maintaining cellular energy homeostasis and is regulated by a network of transcription factors and coactivators [35,36]. MPP^+^ group: MPP^+^ treatment significantly decreased PGC1α (peroxisome proliferator-activated receptor gamma coactivator 1-alpha), NRF1 (nuclear respiratory factor 1), and Tfam (mitochondrial transcription factor A) expression in SH-SY5Y cells (*p* < 0.001), indicating impaired mitochondrial biogenesis and maintenance. RES group: RES-hNSCs-Exos significantly increased PGC1α, NRF1, and Tfam expression compared to the MPP^+^ group (*p* < 0.001), suggesting enhanced mitochondrial biogenesis and improved mitochondrial function. CC group: CC-hNSCs-Exos showed no increase in PGC1α, NRF1, and Tfam gene expression compared with the MPP^+^ group, further supporting the protective role of AMPK in mediating RES-hNSCs-Exos. Figure 4 and Figure 5 illustrate that RES-hNSCs-Exos activate critical mitochondrial biogenesis and function regulators, suggesting their potential to improve mitochondrial health and protect neurons from MPP^+^-induced damage. This highlights the role of mitochondrial support in the neuroprotective effects of these Exos.

### 3.5. hNSCs-Exos Promote Antioxidant Response and Activate Nrf2 in SH-SY5Y Cells Exposed to MPP^+^

**Figure 6:**
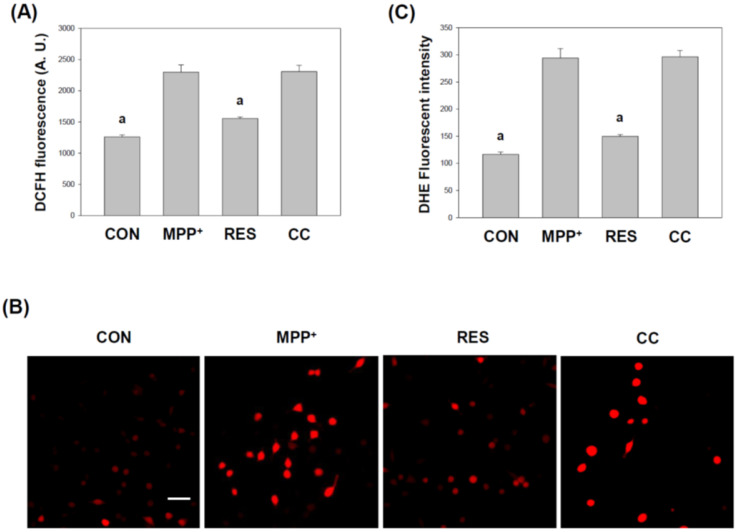
**hNSCs-Exos Normalize** MPP^+^**-Induced Oxidative Stress in SH-SY5Y Cells via the AMPK Pathway.** SH-SY5Y cells were treated with MPP^+^ and RES-hNSCs-Exos to investigate the effects of the Exos on oxidative stress, with a particular focus on the involvement of the AMPK pathway. (**A**) ROS determination: ROS levels in the cells were analyzed using the DCFH-DA assay, providing insight into oxidative stress levels under different treatment conditions. (**B**) DHE staining for ROS detection: Microphotographs of SH-SY5Y cells stained with DHE dye reveal differences in ROS generation. MPP^+^ treatment alone led to a marked increase in red fluorescence, indicating high levels of ROS and oxidative stress. In contrast, treatment with RES-hNSCs-Exos significantly reduced ROS generation, as evidenced by the decrease in red fluorescence. This suggests that the Exos can mitigate oxidative stress induced by MPP^+^. The scale bar in the images represents 100 μm. (**C**) Quantification of ROS levels: the fluorescence intensity of DHE staining was quantitatively measured and normalized to cell numbers, ensuring accuracy by accounting for variations in cell density. The data represent the mean ± SEM from three independent experiments. The designation “a” indicates a significant difference compared to SH-SY5Y cells treated with MPP^+^ alone (*p* < 0.001, one-way ANOVA).

Figure 6 investigates the effect of RES-hNSCs-Exos on reducing oxidative stress in SH-SY5Y cells exposed to MPP^+^-induced neurotoxicity. Oxidative stress is a crucial contributor to neurodegenerative diseases, and the AMPK pathway plays a vital role in regulating cellular energy and mitigating oxidative stress [24,37]. Reactive Oxygen Species (ROS) determination (Panel A): ROS levels were assessed using the DCFH-DA assay, which detects intracellular ROS generation. The data demonstrate a significant increase in ROS production in MPP^+^-treated cells, indicating elevated oxidative stress. However, treatment with RES-hNSCs-Exos significantly reduced ROS levels, suggesting that these Exos alleviate oxidative damage.

Staining for ROS detection (Panel B): Dihydroethidium (DHE) staining was used to visualize ROS production. In MPP^+^-treated cells, there was an increase in red fluorescence, indicating high ROS levels. In contrast, cells treated with RES-hNSCs-Exos showed a substantial reduction in ROS, as demonstrated by diminished red fluorescence. This further confirms the Exos’ ability to lower oxidative stress. Quantification of ROS levels (Panel C): quantitative analysis of the fluorescence intensity from DHE staining confirmed that RES-hNSCs-Exos significantly reduced ROS levels compared to MPP^+^-treated cells. The results highlight the role of the AMPK pathway in the protective effects of these Exos against oxidative stress. CC-hNSCs-Exos showed no improvement in ROS levels compared with the MPP^+^ group (Figure 6), indicating the involvement of the AMPK pathway in reducing oxidative stress. Figure 6 shows that RES-hNSCs-Exos effectively mitigates oxidative stress in SH-SY5Y cells by reducing ROS levels, likely activating the AMPK pathway. This reduction in oxidative stress is crucial for protecting neuronal cells from MPP^+^-induced neurotoxicity, suggesting a potential therapeutic approach for neurodegenerative diseases.

**Figure 7:**
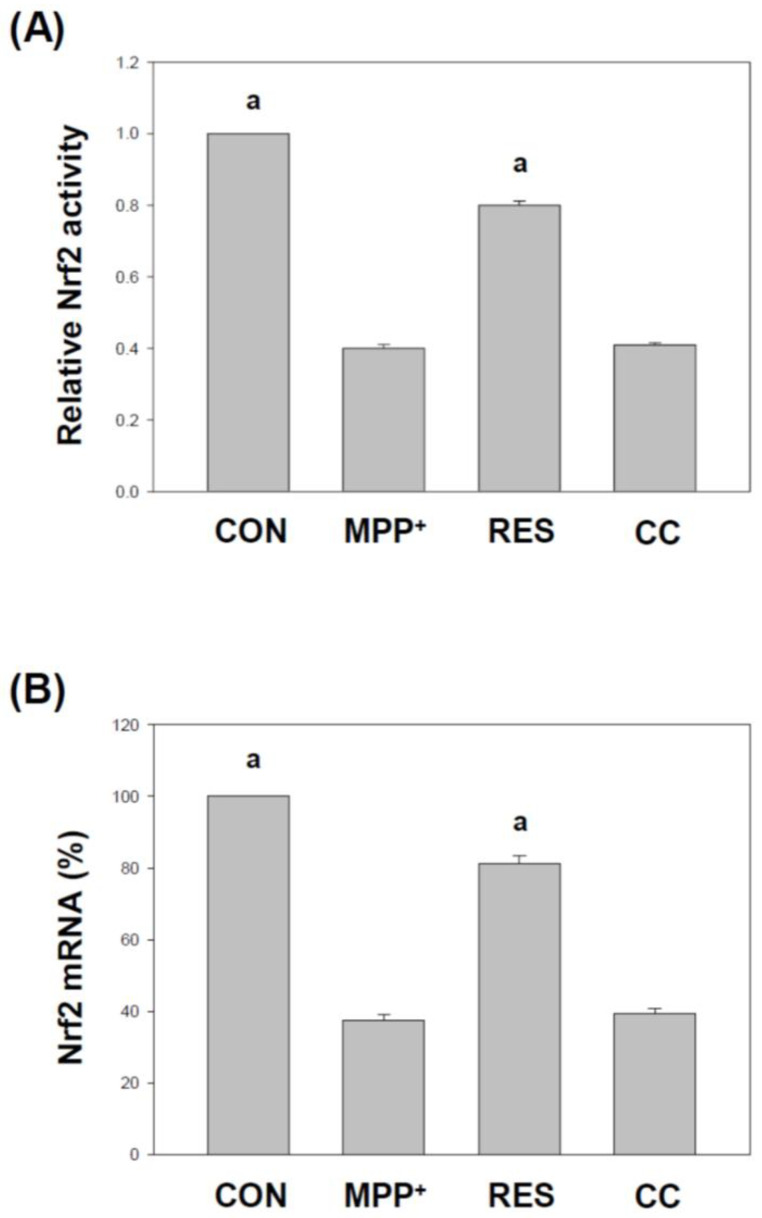
**hNSCs-Exos Increase Nrf2 Activity and Gene Expression in SH-SY5Y Cells Exposed to** MPP^+^**.** SH-SY5Y cells were treated following the protocol described in Figure 1 to evaluate the effects of RES-hNSCs-Exos on the activity and gene expression of Nrf2 in the context of MPP^+^-induced neurotoxicity. (**A**) Nrf2 protein activity: Nrf2 activity in the nucleus was measured using the Nrf2 transcription factor assay kit, which quantifies the active form of Nrf2, reflecting its transcriptional activity. The assay demonstrated a significant increase in Nrf2 activity in cells treated with RES-hNSCs-Exos compared to those treated with MPP^+^ alone. (**B**) Nrf2 gene expression: The mRNA expression levels of Nrf2 were analyzed using qPCR. RNA from SH-SY5Y cells was extracted, reverse transcribed into cDNA, and subjected to qPCR analysis. The results were normalized to GAPDH as the reference gene, with each reaction conducted in triplicate. The data represent mean ± SEM from three independent experiments. The designation “a” indicates a significant difference compared to SH-SY5Y cells treated with MPP^+^ alone (*p* < 0.001, one-way ANOVA).

**Figure 8:**
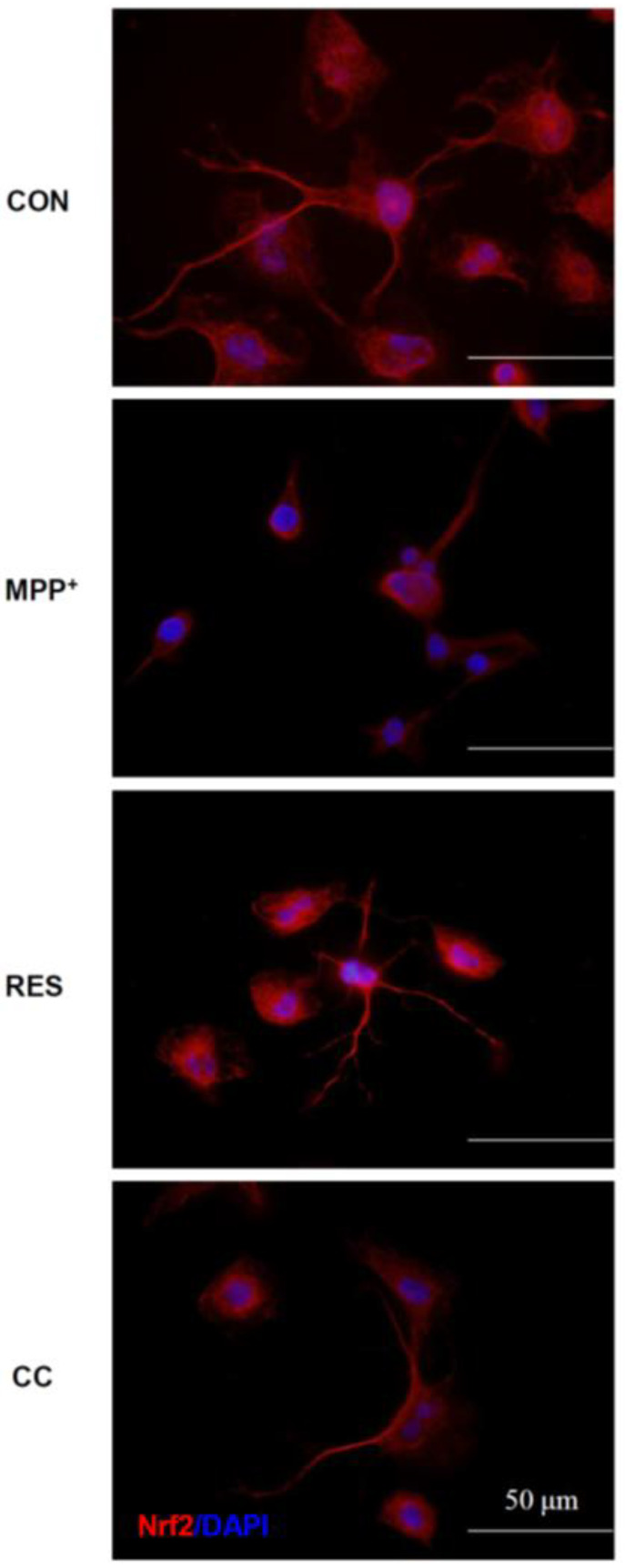
**Effects of hNSCs-Exos on Immunostaining Analysis of Nrf2 Levels in SH-SY5Y Cells Exposed to** MPP^+^**.** Cells were treated following the protocol outlined in Figure 1 to assess Nrf2 protein levels and their cellular localization in SH-SY5Y cells exposed to MPP^+^, with and without RES-hNSCs-Exos. Immunostaining: Antibodies against Nrf2 (red) were used to visualize its expression in SH-SY5Y cells. The nucleus was counterstained with DAPI (blue fluorescence). Representative images show distinct Nrf2 expression patterns across different treatment conditions. Nrf2 levels significantly increased in cells treated with RES-hNSCs-Exos. The scale bar represents 50 μm.

Figure 7 and Figure 8 explore the impact of RES-hNSCs-Exos on the nuclear factor erythroid 2-related factor 2 (Nrf2) signaling pathway in SH-SY5Y cells exposed to MPP^+^-induced neurotoxicity. Nrf2 is a crucial regulator of cellular antioxidant defense mechanisms, and its activation helps protect cells from oxidative stress, a key feature in neurodegenerative diseases [38,39]. Figure 7: Nrf2 activity and gene expression. Panel A (Nrf2 protein activity): Nrf2 activity was measured in the nucleus using a transcription factor assay kit. The results show that RES-hNSCs-Exos significantly increased Nrf2 activity in MPP^+^-treated cells, indicating that these Exos enhance the activation of the Nrf2 pathway, leading to increased cellular defense against oxidative stress. Panel B (Nrf2 gene expression): The expression levels of Nrf2 mRNA were analyzed using qPCR. Cells treated with RES-hNSCs-Exos showed a significant upregulation of Nrf2 gene expression compared to cells treated with MPP^+^ alone. This indicates that the Exos not only increase Nrf2 activity, but also enhance its gene transcription, further amplifying the antioxidant response. Figure 8: Immunostaining analysis of Nrf2 levels. Immunofluorescence staining was used to visualize Nrf2 expression and subcellular localization. In MPP^+^-treated cells, Nrf2 levels were low. However, treatment with RES-hNSCs-Exos led to a marked increase in Nrf2 expression, where it exerts its protective functions. The enhanced localization of Nrf2 suggests that the Exos promote its activation. Quantitative analysis (Figure S2) of the immunostaining results confirmed a significant increase in Nrf2 levels in cells treated with RES-hNSCs-Exos, compared to MPP^+^ alone. This reinforces the observation that these Exos potentiate the Nrf2-mediated antioxidant defense. CC-hNSCs-Exos showed no increase in Nrf2 expression compared with the MPP^+^ group (Figure 7, Figure 8 and Figure S2), further supporting the involvement of the AMPK pathway in upregulating Nrf2 expression, contributing to the cell’s antioxidant defense system. Figure 7, Figure 8 and Figure S2 demonstrate that RES-hNSCs-Exos activate the Nrf2 pathway in SH-SY5Y cells, increasing Nrf2 activity and expression. This activation is crucial for enhancing the cellular antioxidant response, which helps counteract the oxidative stress caused by MPP^+^-induced neurotoxicity. These findings underscore the potential of hNSCs-Exos as a neuroprotective therapeutic strategy in neurodegenerative diseases.

### 3.6. hNSCs-Exos Suppress Inflammasome Activation by Reducing NLRP3 Expression in SH-SY5Y Cells Exposed to MPP^+^

**Figure 9:**
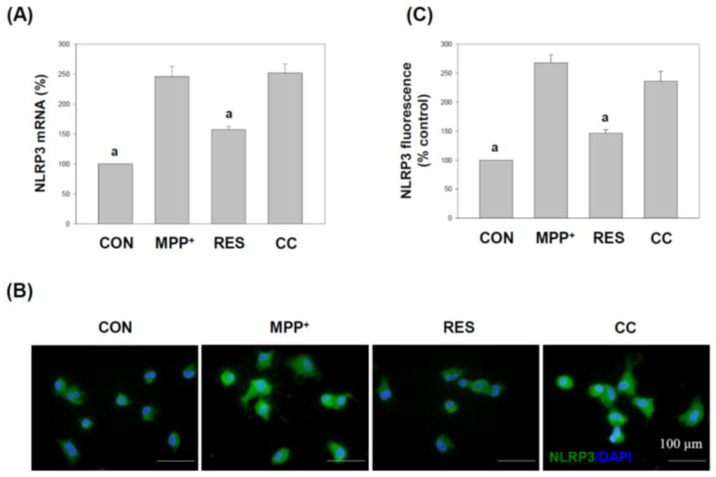
Effects of **hNSCs-Exos** on NLRP3 Expression in SH-SY5Y Cells Exposed to MPP^+^. (**A**) mRNA expression analysis: the mRNA transcripts of NLRP3 were analyzed using qPCR. RNA was collected from SH-SY5Y cells, reverse transcribed into cDNA, and subjected to qPCR to determine NLRP3 expression levels. (**B**) Immunostaining: Cells were stained with antibodies specific to NLRP3, visualized with avidin–Alexa Fluor^®^ 488-conjugated secondary antibody (green fluorescence), and counterstained with DAPI (blue) to mark nuclei. Representative images from three independent experiments are shown. Scale bar: 100 μm. (**C**) Quantification of fluorescence intensity: The fluorescence intensity of NLRP3 expression was quantified from the immunostaining results. Green fluorescence represents NLRP3 expression, and a statistical analysis of fluorescence intensity was performed. The data represent mean ± SEM from three independent experiments. S The designation “a” indicates a significant difference compared to SH-SY5Y cells treated with MPP^+^ alone (*p* < 0.001, one-way ANOVA).

Figure 9 shows the effects of RES-hNSCs-Exos on the expression of NLRP3 in SH-SY5Y cells exposed to MPP^+^-induced neurotoxicity. NLRP3 is a critical component of the NLRP3 inflammasome, which plays a crucial role in regulating inflammatory responses, particularly in neurodegenerative diseases where inflammation contributes to neuronal damage [40,41]. Panel A (NLRP3 mRNA expression analysis): The mRNA expression levels of NLRP3 were measured using qPCR. In SH-SY5Y cells treated with MPP^+^, NLRP3 expression was significantly upregulated, indicating the activation of the inflammasome and an increase in inflammatory signaling. However, treatment with RES-hNSCs-Exos significantly reduced NLRP3 mRNA levels, suggesting that the Exos inhibit inflammasome activation and help mitigate inflammation in response to MPP^+^-induced stress.

Panel B (immunostaining for NLRP3 expression): Immunofluorescence staining was employed to visualize NLRP3 protein levels in the treated SH-SY5Y cells. Cells exposed to MPP^+^ exhibited increased green fluorescence, indicating high NLRP3 expression. In contrast, cells treated with RES-hNSCs-Exos showed a marked reduction in NLRP3 expression, visually confirming the inhibitory effect of the Exos on NLRP3-mediated inflammatory responses. Panel C (quantification of fluorescence intensity): Quantitative immunostaining analysis confirmed a significant decrease in NLRP3 expression in cells treated with RES-hNSCs-Exos compared to MPP^+^-treated cells. This reduction highlights the ability of the Exos to downregulate the expression of NLRP3, a key mediator of neuroinflammation. CC-hNSCs-Exos showed no decrease in NLRP3 expression compared with the MPP^+^ group, further supporting the involvement of the AMPK pathway in inhibiting inflammasome activation and mitigating inflammatory responses. Figure 9 demonstrates that RES-hNSCs-Exos effectively reduce the expression of NLRP3 in SH-SY5Y cells exposed to MPP^+^, both at the mRNA and protein levels. This suggests that these Exos have anti-inflammatory properties, potentially reducing inflammasome activation and mitigating neuroinflammation, a hallmark of neurodegenerative diseases.

### 3.7. Effects of hNSCs-Exos Reduce ASC, Caspase-1 Expression, and Pro-Inflammatory Cytokine Secretion in SH-SY5Y Cells Exposed to MPP^+^

**Figure 10:**
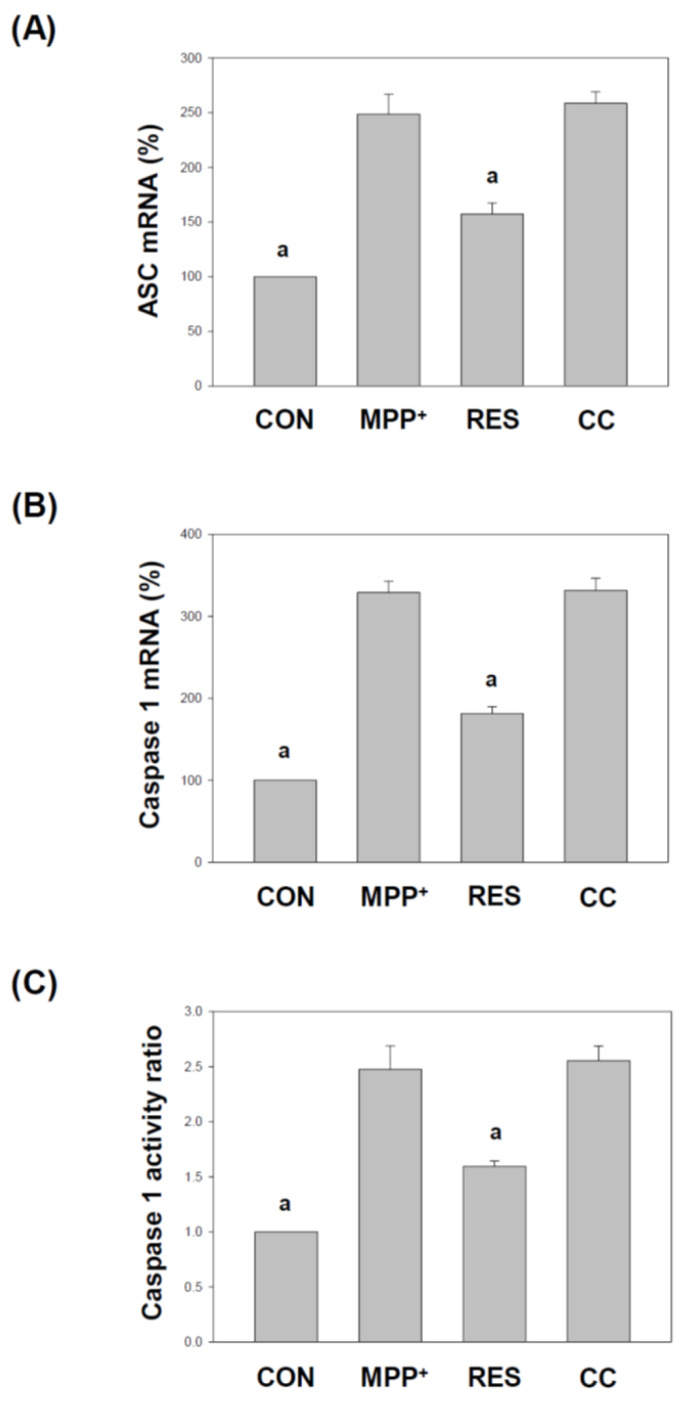
Effects of **hNSCs-Exos** on ASC and Caspase-1 Expression and Activity in SH-SY5Y Cells Exposed to MPP^+^. (**A**) ASC mRNA expression analysis: The mRNA transcripts of ASC were measured using qPCR. RNA was extracted from SH-SY5Y cells, reverse transcribed into cDNA, and subjected to qPCR to determine ASC expression levels. (**B**) Caspase-1 mRNA expression analysis: the mRNA transcripts of caspase-1 were also analyzed using qPCR following the same protocol as ASC to measure its expression. (**C**) Caspase-1 activity analysis: The activity of caspase-1 was measured using a Caspase-Glo^®^ 1 inflammasome assay kit. Caspase-1 activity was quantified in SH-SY5Y cells to assess the inflammasome activation level. The data represent mean ± SEM from three independent experiments. The designation “a” indicates a significant difference compared to SH-SY5Y cells treated with MPP^+^ alone (*p* < 0.001, one-way ANOVA).

**Figure 11:**
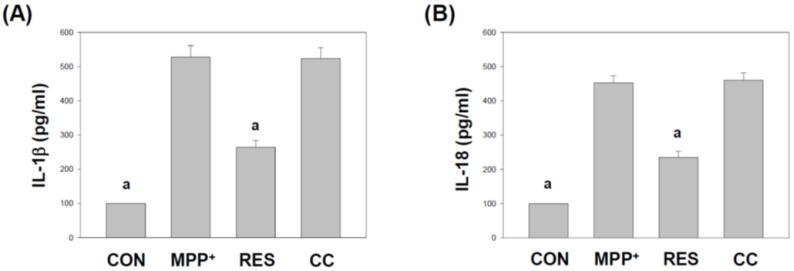
Effects of **hNSCs-Exos** on the Secretion of Inflammatory Cytokines IL-1β and IL-18 in SH-SY5Y Cells Exposed to MPP^+^. (**A**) IL-1β Secretion analysis: The secretion of IL-1β from SH-SY5Y cells was measured using an ELISA assay. Cell culture supernatants were collected following treatment with MPP^+^ and RES-hNSCs-Exos, and IL-1β levels were quantified. (**B**) IL-18 secretion analysis: similarly, the secretion of IL-18 was analyzed using ELISA from the same cell culture supernatants to assess inflammasome-induced cytokine release. The data represent mean ± SEM from three independent experiments. The designation “a” indicates a significant difference compared to SH-SY5Y cells treated with MPP^+^ alone (*p* < 0.001, one-way ANOVA).

Figure 10 and Figure 11 focus on the effects of RES-hNSCs-Exos on inflammasome components and the secretion of inflammatory cytokines in SH-SY5Y cells exposed to MPP^+^-induced neurotoxicity. These figures highlight the role of inflammasome activation in neuroinflammation and how hNSCs-Exos can modulate critical factors involved in this process. ASC (apoptosis-associated speck-like protein) is an essential adaptor protein involved in inflammasome formation [42,43]. Figure 10A: Using qPCR, the mRNA expression levels of ASC were significantly increased in MPP^+^-treated cells, indicating inflammasome activation. However, treatment with RES-hNSCs-Exos significantly reduced ASC expression, suggesting that the Exos can inhibit the activation of the inflammasome pathway. Caspase-1 is an enzyme critical for activating pro-inflammatory cytokines like IL-1β and IL-18 [44]. Figure 10B: Like ASC, caspase-1 mRNA levels were elevated in MPP^+^-treated cells, reflecting inflammasome activation. Treatment with RES-hNSCs-Exos notably reduced caspase-1 expression, suppressing this inflammatory pathway. Figure 10C: Caspase-1 activity, measured using a Caspase-Glo^®^ 1 inflammasome assay, was significantly increased in MPP^+^-treated cells. However, RES-hNSCs-Exos treatment reduced caspase-1 activity, further supporting the anti-inflammatory role of these Exos in inhibiting inflammasome-mediated caspase activation.

IL-1β is a pro-inflammatory cytokine released following inflammasome activation [45]. Figure 11A: In MPP^+^-treated cells, the secretion of IL-1β was significantly increased, indicating an inflammatory response. Treatment with RES-hNSCs-Exos notably reduced IL-1β secretion, suggesting the Exos’ potential to mitigate inflammation. IL-18 is another cytokine processed by caspase-1 during inflammasome activation [44]. Figure 11B: Like IL-1β, IL-18 levels were elevated in MPP^+^-treated cells. Treatment with RES-hNSCs-Exos significantly lowered IL-18 secretion, demonstrating the Exos’ ability to suppress inflammation. CC-hNSCs-Exos showed no reduction in inflammasome pathway and inflammatory cytokines compared with the MPP^+^ group, further supporting the involvement of the AMPK pathway in suppressing inflammasome assembly and mitigating inflammatory cytokine release. Figure 10 and Figure 11 demonstrate that RES-hNSCs-Exos effectively downregulate critical inflammasome components, such as ASC and caspase-1 while reducing the secretion of pro-inflammatory cytokines IL-1β and IL-18. These findings suggest that RES-hNSCs-Exos can suppress neuroinflammation by inhibiting inflammasome activation, thereby providing a potential therapeutic strategy to alleviate MPP^+^-induced neurotoxicity.

## 4. Discussion

RES-hNSCs-Exos significantly rescue SH-SY5Y cells from MPP^+^-induced neurotoxicity by increasing cell viability and reducing apoptosis (Figure 1, Figure 2 and Figure S1). RES-hNSCs-Exos provide moderate protection, indicating the involvement of AMPK in enhancing the neuroprotective effects of hNSC-Exos. RES-hNSCs-Exos significantly increase the expression of AMPK, Bcl-2, and CREB in SH-SY5Y cells exposed to MPP^+^ neurotoxicity, restoring critical survival pathways (Figure 3). RES-hNSCs-Exos significantly increased ATP levels and mitochondrial mass in SH-SY5Y cells exposed to MPP^+^, enhancing cellular energy metabolism and mitochondrial function (Figure 4). RES-hNSCs-Exos significantly upregulated the expression of PGC1α, NRF1, and Tfam in SH-SY5Y cells exposed to MPP^+^, reflecting enhanced mitochondrial biogenesis, gene regulation, and DNA replication (Figure 5). This indicates a robust neuroprotective effect against MPP^+^-induced mitochondrial dysfunction. RES-hNSCs-Exos significantly reduced MPP^+^-induced oxidative stress in SH-SY5Y cells, as evidenced by decreased ROS levels and red fluorescence intensity in DHE staining (Figure 6). These findings suggest that activating the AMPK pathway is critical for the Exos-mediated neuroprotection against MPP^+^-induced oxidative stress. RES-hNSCs-Exos significantly increased the activity and expression of Nrf2 in SH-SY5Y cells under MPP^+^-induced neurotoxic conditions (Figure 7, Figure 8 and Figure S2). This indicates that the Exos are crucial in enhancing the Nrf2 pathway, facilitating expression and activation of Nrf2 to counter oxidative stress, and protecting the cells from MPP^+^-induced neurotoxicity. RES-hNSCs-Exos significantly downregulated both mRNA and protein levels of NLRP3 in SH-SY5Y cells exposed to MPP^+^, effectively reducing the activation of the NLRP3 inflammasome (Figure 9). This indicates that the Exos provide anti-inflammatory protection, mitigating the neuroinflammatory response induced by MPP^+^-mediated neurotoxicity. RES-hNSCs-Exos effectively inhibit the activation of the inflammasome pathway by reducing ASC and caspase-1 expression and activity, as well as decreasing the secretion of inflammatory cytokines IL-1β and IL-18 in SH-SY5Y cells exposed to MPP^+^ (Figure 10 and Figure 11). These findings demonstrate the Exos’ potential to mitigate MPP^+^-induced neuroinflammation and neurotoxicity. This study extensively evaluated the neuroprotective potential of RES-hNSCs-Exos in mitigating MPP^+^-induced neurotoxicity in SH-SY5Y cells. The findings highlight multiple mechanisms through which these Exos exert protective effects, emphasizing their role in cell survival, mitochondrial preservation, and anti-inflammatory responses, all relevant to neurodegenerative conditions like PD.

In recent years, Exos, nanoscale extracellular vesicles secreted by various cell types, have emerged as potential therapeutic agents due to their ability to transfer bioactive molecules, such as proteins, lipids, and microRNAs, between cells [46,47]. Given their regenerative potential, hNSCs represent a promising source of Exos for neuroprotection and neuroregeneration in neurodegenerative diseases like PD [16,48]. When hNSCs are treated with RES, they may produce Exos enriched with bioactive molecules—including proteins, microRNAs, and other signaling molecules—that amplify the neuroprotective potential of these Exos. The RES-enhanced Exos are expected to modulate oxidative stress and inflammation better, protect neuronal mitochondria, and reduce apoptosis when delivered to target cells, such as SH-SY5Y cells. This enhanced therapeutic capability makes these Exos a promising tool for mitigating neurodegenerative damage, particularly in PD models. RES-hNSCs-Exos delivery has advantages over direct administration. Although RES has known neuroprotective effects, its bioavailability, stability, and cellular uptake are major limiting factors in vivo [49,50]. RES-hNSCs-Exos can enhance the therapeutic effect of RES in the following ways. RES-hNSCs-Exos improve stability and bioavailability, facilitate more efficient crossing of biological barriers (such as the blood–brain barrier; BBB), and enhance cellular uptake. hNSC-derived Exos also contain additional neuroprotective factors that may improve the beneficial effects of RES [51,52].

The results presented in Figure 1, Figure 2, Figure 3, Figure 4, Figure 5, Figure 6, Figure 7, Figure 8, Figure 9, Figure 10 and Figure 11 provide significant insights into the neuroprotective effects of RES-hNSCs-Exos on SH-SY5Y cells exposed to MPP^+^-induced neurotoxicity, a widely used model of PD. The findings underscore the role of hNSCs-Exos in mitigating oxidative stress, mitochondrial dysfunction, and neuroinflammation through various signaling pathways, offering a promising therapeutic strategy for neurodegenerative diseases like PD. Figure 1 and Figure 2 demonstrate that MPP^+^ treatment significantly reduces SH-SY5Y cell viability, indicating severe neurotoxic stress. However, upon treatment with RES-hNSCs-Exos, cell viability markedly improves, as higher survival rates show. These findings suggest that hNSCs-Exos effectively counteract the cytotoxic effects of MPP^+^, potentially preventing neuronal cell death, which is a critical factor in PD pathogenesis. This underscores the therapeutic potential of hNSCs-Exos in protecting against neurodegeneration [53,54]. hNSCs-Exos activate the AMPK pathway, a key regulator of cellular stress responses, promoting the expression of anti-apoptotic markers like Bcl-2 and CREB. These findings suggest that Exos enhance cellular survival under oxidative stress by reducing apoptosis, effectively protecting neurons from programmed cell death in PD. As shown in Figure 3, hNSCs-Exos activated the AMPK pathway, a crucial cellular energy sensor that regulates mitochondrial homeostasis and stress responses. AMPK: A master regulator of energy homeostasis, AMPK activation is vital for cellular survival under stress conditions, such as those induced by MPP^+^. Increased expression of AMPK suggests enhanced cellular energy metabolism and protection against energy depletion [55,56]. Bcl-2 is an anti-apoptotic gene that plays a vital role in preventing programmed cell death by inhibiting the mitochondrial apoptosis pathway [57]. Its increased expression indicates the ability of hNSCs-Exos to reduce apoptosis in SH-SY5Y cells exposed to neurotoxic stress. CREB is a transcription factor involved in neuronal plasticity, survival, and neuroprotection [58,59]. Increased CREB expression suggests that hNSCs-Exos enhances cellular resilience to promote neuroprotective signaling pathways. The upregulation of AMPK and the anti-apoptotic markers Bcl-2 and CREB in SH-SY5Y cells suggests that hNSCs-Exos promote cell survival and further supports this anti-apoptotic mechanism.

Mitochondrial dysfunction is a hallmark of MPP^+^-induced neurotoxicity, as evidenced by decreased ATP levels and disrupted mitochondrial morphology [7,60]. Figure 4 highlights the beneficial effects of hNSCs-Exos on mitochondrial function, a critical factor in PD pathology. MPP^+^ treatment, known to induce mitochondrial dysfunction, was mitigated by RES-hNSCs-Exos. This increased cellular ATP production and mitochondrial mass, as demonstrated through ATP assays and Mitotracker Green™ staining. This suggests that hNSCs-Exos boost mitochondrial biogenesis and energy metabolism, aiding cells in managing MPP^+^-induced stress. hNSCs-Exos promote mitochondrial biogenesis via the PGC1α/NRF1/Tfam pathway. As shown in Figure 5, hNSCs-Exos significantly upregulated the expression of PGC1α, NRF1, and Tfam, critical regulators of mitochondrial biogenesis. PGC1α acts as a master regulator that activates genes involved in mitochondrial function [61]. At the same time, NRF1 supports the transcription of mitochondrial respiratory genes, and Tfam is essential for mitochondrial DNA replication and maintenance [62,63]. This upregulation indicates that hNSCs-Exos actively promote mitochondrial biogenesis, allowing cells to generate new mitochondria to counteract the harmful effects of MPP^+^-induced mitochondrial dysfunction, thereby improving overall mitochondrial health and energy production.

- The generation of ROS is a critical factor in MPP^+^-induced cell death [64,65]. hNSCs-Exos demonstrated potent antioxidative effects by significantly reducing ROS levels, as evidenced by the DCFH-DA assay and DHE staining (Figure 6). The involvement of the AMPK pathway in this process is crucial, as AMPK activation enhances antioxidant defenses [37,66]. This ROS reduction can be attributed to the activation of the Nrf2 signaling pathway, an essential regulator of the cellular antioxidant response [67]. The Nrf2 pathway is crucial for regulating the expression of antioxidant genes, and Figure 7 and Figure 8 reveal that hNSCs-Exos significantly enhance Nrf2 activity and gene expression in SH-SY5Y cells. Treatment with RES-hNSCs-Exos activated the Nrf2 pathway, as immunostaining shows. By boosting Nrf2 activity, hNSCs-Exos promote the expression of antioxidant genes, strengthening the cell’s defense against oxidative stress and helping restore redox balance in cells exposed to MPP^+^-induced neurotoxicity. This suggests that the neuroprotective effects of hNSCs-Exos are mediated, at least in part, by the upregulation of antioxidant defenses via Nrf2 activation. Although Compound C was used as an AMPK inhibitor to confirm the pathway’s involvement, this study did not include inhibitors targeting downstream pathways, such as Nrf2. Previous literature supports a strong interaction between AMPK and Nrf2, indicating that AMPK activation can modulate Nrf2 activity [68,69]. Thus, inhibiting AMPK may provide indirect but significant insights into the regulation of Nrf2. This study focused on how AMPK activation enhances mitochondrial function and mitigates oxidative stress and neuroinflammation. However, exploring Nrf2 remains a promising direction for future research. Oxidative stress is a major contributor to neurodegenerative diseases, and its interplay with protein homeostasis is crucial for disease progression [70]. Excessive oxidative stress impairs the function of protein degradation systems, such as the ubiquitin-proteasome system (UPS) and the autophagy-lysosomal pathway, leading to the accumulation of misfolded proteins—a hallmark of PD [71,72]. RES-hNSCs-Exos-based therapy may help restore protein homeostasis by mitigating oxidative stress. This preserves protein degradation mechanisms and prevents protein aggregation. A significant challenge in using Exos to treat neurodegenerative diseases is their ability to cross the BBB [14,53]. RES-hNSCs-Exos offer a promising multi-target neuroprotective strategy for PD; however, ensuring effective delivery in vivo is crucial for future clinical applications. This study was designed as an in vitro experiment, focusing primarily on cellular neuroprotection and its underlying mechanisms. While we recognize the importance of BBB permeability, further in vivo investigations are necessary to address this aspect.
- Neuroinflammation is a pivotal component of PD pathology, often exacerbated by the activation of the NLRP3 inflammasome [12,73,74]. Figure 9 illustrates that treatment with RES-hNSCs-Exos significantly reduced NLRP3 expression in MPP^+^-treated SH-SY5Y cells, as both qPCR and immunostaining analyses show. The downregulation of NLRP3 expression suggests that hNSCs-Exos can inhibit inflammasome activation, potentially mitigating the neuroinflammatory processes that lead to progressive neuronal damage in PD. Further supporting the anti-inflammatory role of hNSCs-Exos, Figure 10 and Figure 11 demonstrate that Exos treatment downregulated the expression of inflammasome components ASC and caspase-1 while reducing caspase-1 activity. Caspase-1 was measured independently due to its distinct role in inflammasome activation and neuroinflammation [69,74]. In contrast, caspase-3 and caspase-9 were assessed to evaluate apoptotic pathways [75,76]. Since inflammasome activation and apoptosis are related but mechanistically distinct processes, analyzing caspase-1 separately allowed for a more apparent distinction between neuroinflammatory and apoptotic responses. The reduction of caspase-1 activity is significant, as this enzyme mediates the cleavage and activation of pro-inflammatory cytokines such as IL-1β and IL-18. ELISA assays showed that hNSCs-Exos significantly decreased the secretion of IL-1β and IL-18, critical mediators of neuroinflammation. These findings suggest that hNSCs-Exos inhibit the inflammasome, reducing the release of pro-inflammatory cytokines and mitigating inflammation-related neuronal damage. This anti-inflammatory effect is crucial for mitigating neurodegenerative processes, as chronic inflammation can exacerbate neuronal damage and contribute to disease progression.

In conclusion, RES-hNSCs-Exos confer significant neuroprotection against MPP^+^-induced neurotoxicity in SH-SY5Y cells. These Exos preserve cell viability, enhance mitochondrial function, mitigate oxidative stress, and suppress inflammasome activation. The activation of the AMPK and Nrf2 pathways appears to be critical in mediating these protective effects. Given the multifaceted roles of hNSC-Exos in neuroprotection, hNSC-Exos represent a promising therapeutic tool for treating neurodegenerative diseases such as PD by targeting multiple mechanisms involved in neuronal health and survival.

## Data Availability

Data will be made available on request.

## Supplementary Materials

The following supporting information can be downloaded at https://www.mdpi.com/article/10.3390/life15020294/s1: Figure S1: Effects of hNSCs-Exos on Immunostaining Analysis of Caspase 3 Levels in SH-SY5Y Cells Exposed to MPP^+^; Figure S2: Effects of hNSCs-Exos on Nrf2 Levels in SH-SY5Y Cells Exposed to MPP^+^.

## References

[1] Pang M., Peng R., Wang Y., Zhu Y., Wang P., Moussian B., Su Y., Liu X., Ming D. (2022). Molecular understanding of the translational models and the therapeutic potential natural products of Parkinson’s disease. Biomed. Pharmacother..

[2] Morris H.R., Spillantini M.G., Sue C.M., Williams-Gray C.H. (2024). The pathogenesis of Parkinson’s disease. Lancet.

[3] Schalkamp A.K., Peall K.J., Harrison N.A., Sandor C. (2023). Wearable movement-tracking data identify Parkinson’s disease years before clinical diagnosis. Nat. Med..

[4] Opara J., Malecki A., Malecka E., Socha T. (2017). Motor assessment in Parkinson;s disease. Ann. Agric. Environ. Med..

[5] Yen C., Lin C.L., Chiang M.C. (2023). Exploring the Frontiers of Neuroimaging: A Review of Recent Advances in Understanding Brain Functioning and Disorders. Life.

[6] Gao X.Y., Yang T., Gu Y., Sun X.H. (2022). Mitochondrial Dysfunction in Parkinson’s Disease: From Mechanistic Insights to Therapy. Front. Aging Neurosci..

[7] Henrich M.T., Oertel W.H., Surmeier D.J., Geibl F.F. (2023). Mitochondrial dysfunction in Parkinson’s disease—A key disease hallmark with therapeutic potential. Mol. Neurodegener..

[8] Hemmati-Dinarvand M., Saedi S., Valilo M., Kalantary-Charvadeh A., Alizadeh Sani M., Kargar R., Safari H., Samadi N. (2019). Oxidative stress and Parkinson’s disease: Conflict of oxidant-antioxidant systems. Neurosci. Lett..

[9] Chiang M.C., Tsai T.Y., Wang C.J. (2023). The Potential Benefits of Quercetin for Brain Health: A Review of Anti-Inflammatory and Neuroprotective Mechanisms. Int. J. Mol. Sci..

[10] Dong-Chen X., Yong C., Yang X., Chen-Yu S., Li-Hua P. (2023). Signaling pathways in Parkinson’s disease: Molecular mechanisms and therapeutic interventions. Signal Transduct. Target. Ther..

[11] Xiao B., Kuruvilla J., Tan E.K. (2022). Mitophagy and reactive oxygen species interplay in Parkinson’s disease. NPJ Park. Dis..

[12] Nguyen L.T.N., Nguyen H.D., Kim Y.J., Nguyen T.T., Lai T.T., Lee Y.K., Ma H.I., Kim Y.E. (2022). Role of NLRP3 Inflammasome in Parkinson’s Disease and Therapeutic Considerations. J. Park. Dis..

[13] Saikia B., Dhanushkodi A. (2024). Engineered exosome therapeutics for neurodegenerative diseases. Life Sci..

[14] Nouri Z., Barfar A., Perseh S., Motasadizadeh H., Maghsoudian S., Fatahi Y., Nouri K., Yektakasmaei M.P., Dinarvand R., Atyabi F. (2024). Exosomes as therapeutic and drug delivery vehicle for neurodegenerative diseases. J. Nanobiotechnol..

[15] Bonetto V., Grilli M. (2023). Neural stem cell-derived extracellular vesicles: Mini players with key roles in neurogenesis, immunomodulation, neuroprotection and aging. Front. Mol. Biosci..

[16] Zhang G., Zhu Z., Wang H., Yu Y., Chen W., Waqas A., Wang Y., Chen L. (2020). Exosomes derived from human neural stem cells stimulated by interferon gamma improve therapeutic ability in ischemic stroke model. J. Adv. Res..

[17] Zhong L., Wang J., Wang P., Liu X., Liu P., Cheng X., Cao L., Wu H., Chen J., Zhou L. (2023). Neural stem cell-derived exosomes and regeneration: Cell-free therapeutic strategies for traumatic brain injury. Stem Cell Res. Ther..

[18] Koushki M., Amiri-Dashatan N., Ahmadi N., Abbaszadeh H.A., Rezaei-Tavirani M. (2018). Resveratrol: A miraculous natural compound for diseases treatment. Food Sci. Nutr..

[19] Meng T., Xiao D., Muhammed A., Deng J., Chen L., He J. (2021). Anti-Inflammatory Action and Mechanisms of Resveratrol. Molecules.

[20] Chiang M.C., Nicol C.J., Cheng Y.C. (2018). Resveratrol activation of AMPK-dependent pathways is neuroprotective in human neural stem cells against amyloid-beta-induced inflammation and oxidative stress. Neurochem. Int..

[21] Wicinski M., Erdmann J., Nowacka A., Kuzminski O., Michalak K., Janowski K., Ohla J., Biernaciak A., Szambelan M., Zabrzynski J. (2023). Natural Phytochemicals as SIRT Activators-Focus on Potential Biochemical Mechanisms. Nutrients.

[22] Curry D.W., Stutz B., Andrews Z.B., Elsworth J.D. (2018). Targeting AMPK Signaling as a Neuroprotective Strategy in Parkinson’s Disease. J. Park. Dis..

[23] Sharma A., Anand S.K., Singh N., Dwivedi U.N., Kakkar P. (2023). AMP-activated protein kinase: An energy sensor and survival mechanism in the reinstatement of metabolic homeostasis. Exp. Cell Res..

[24] Zhao Z., Yan J., Huang L., Yang X. (2024). Phytochemicals targeting Alzheimer’s disease via the AMP-activated protein kinase pathway, effects, and mechanisms of action. Biomed. Pharmacother..

[25] Liao Z., Gong Z., Wang Z., Yang W., Liu W., Hou L., Liu X., Hua J., Wang B., Li N. (2022). The Degradation of TMEM166 by Autophagy Promotes AMPK Activation to Protect SH-SY5Y Cells Exposed to MPP^+^. Cells.

[26] Lin C.H., Nicol C.J.B., Cheng Y.C., Yen C., Wang Y.S., Chiang M.C. (2020). Neuroprotective effects of resveratrol against oxygen glucose deprivation induced mitochondrial dysfunction by activation of AMPK in SH-SY5Y cells with 3D gelatin scaffold. Brain Res..

[27] Bae J.E., Kim J.B., Jo D.S., Park N.Y., Kim Y.H., Lee H.J., Kim S.H., Kim S.H., Son M., Kim P. (2022). Carnitine Protects against MPP(+)-Induced Neurotoxicity and Inflammation by Promoting Primary Ciliogenesis in SH-SY5Y Cells. Cells.

[28] Jung Y.J., Choi H., Oh E. (2021). Effects of particulate matter and nicotine for the MPP+-induced SH-SY5Y cells: Implication for Parkinson’s disease. Neurosci. Lett..

[29] Kim H.Y., Jeon H., Kim H., Koo S., Kim S. (2018). Sophora flavescens Aiton Decreases MPP(+)-Induced Mitochondrial Dysfunction in SH-SY5Y Cells. Front. Aging Neurosci..

[30] Xicoy H., Wieringa B., Martens G.J. (2017). The SH-SY5Y cell line in Parkinson’s disease research: A systematic review. Mol. Neurodegener..

[31] Lin C.H., Nicol C.J.B., Wan C., Chen S.J., Huang R.N., Chiang M.C. (2022). Exposure to PM(2.5) induces neurotoxicity, mitochondrial dysfunction, oxidative stress and inflammation in human SH-SY5Y neuronal cells. Neurotoxicology.

[32] Prasertsuksri P., Kraokaew P., Pranweerapaiboon K., Sobhon P., Chaithirayanon K. (2023). Neuroprotection of Andrographolide against Neurotoxin MPP(+)-Induced Apoptosis in SH-SY5Y Cells via Activating Mitophagy, Autophagy, and Antioxidant Activities. Int. J. Mol. Sci..

[33] Klemmensen M.M., Borrowman S.H., Pearce C., Pyles B., Chandra B. (2024). Mitochondrial dysfunction in neurodegenerative disorders. Neurotherapeutics.

[34] Bustamante-Barrientos F.A., Luque-Campos N., Araya M.J., Lara-Barba E., de Solminihac J., Pradenas C., Molina L., Herrera-Luna Y., Utreras-Mendoza Y., Elizondo-Vega R. (2023). Mitochondrial dysfunction in neurodegenerative disorders: Potential therapeutic application of mitochondrial transfer to central nervous system-residing cells. J. Transl. Med..

[35] Liu L., Li Y., Chen G., Chen Q. (2023). Crosstalk between mitochondrial biogenesis and mitophagy to maintain mitochondrial homeostasis. J. Biomed. Sci..

[36] Zong Y., Li H., Liao P., Chen L., Pan Y., Zheng Y., Zhang C., Liu D., Zheng M., Gao J. (2024). Mitochondrial dysfunction: Mechanisms and advances in therapy. Signal Transduct. Target. Ther..

[37] Marino A., Hausenloy D.J., Andreadou I., Horman S., Bertrand L., Beauloye C. (2021). AMP-activated protein kinase: A remarkable contributor to preserve a healthy heart against ROS injury. Free Radic. Biol. Med..

[38] George M., Tharakan M., Culberson J., Reddy A.P., Reddy P.H. (2022). Role of Nrf2 in aging, Alzheimer’s and other neurodegenerative diseases. Ageing Res. Rev..

[39] Mayer C., Riera-Ponsati L., Kauppinen S., Klitgaard H., Erler J.T., Hansen S.N. (2024). Targeting the NRF2 pathway for disease modification in neurodegenerative diseases: Mechanisms and therapeutic implications. Front. Pharmacol..

[40] Chen Y., Ye X., Escames G., Lei W., Zhang X., Li M., Jing T., Yao Y., Qiu Z., Wang Z. (2023). The NLRP3 inflammasome: Contributions to inflammation-related diseases. Cell Mol. Biol. Lett..

[41] Tao S., Fan W., Liu J., Wang T., Zheng H., Qi G., Chen Y., Zhang H., Guo Z., Zhou F. (2023). NLRP3 Inflammasome: An Emerging Therapeutic Target for Alzheimer’s Disease. J. Alzheimers Dis..

[42] Prather E.R., Gavrilin M.A., Wewers M.D. (2022). The central inflammasome adaptor protein ASC activates the inflammasome after transition from a soluble to an insoluble state. J. Biol. Chem..

[43] Yao J., Sterling K., Wang Z., Zhang Y., Song W. (2024). The role of inflammasomes in human diseases and their potential as therapeutic targets. Signal Transduct. Target. Ther..

[44] Zheng D., Liwinski T., Elinav E. (2020). Inflammasome activation and regulation: Toward a better understanding of complex mechanisms. Cell Discov..

[45] Trachalaki A., Tsitoura E., Mastrodimou S., Invernizzi R., Vasarmidi E., Bibaki E., Tzanakis N., Molyneaux P.L., Maher T.M., Antoniou K. (2021). Enhanced IL-1beta Release Following NLRP3 and AIM2 Inflammasome Stimulation Is Linked to mtROS in Airway Macrophages in Pulmonary Fibrosis. Front. Immunol..

[46] Chen Y.F., Luh F., Ho Y.S., Yen Y. (2024). Exosomes: A review of biologic function, diagnostic and targeted therapy applications, and clinical trials. J. Biomed. Sci..

[47] Abdulmalek O., Husain K.H., AlKhalifa H., Alturani M., Butler A.E., Moin A.S.M. (2024). Therapeutic Applications of Stem Cell-Derived Exosomes. Int. J. Mol. Sci..

[48] Cecerska-Heryc E., Pekala M., Serwin N., Glizniewicz M., Grygorcewicz B., Michalczyk A., Heryc R., Budkowska M., Dolegowska B. (2023). The Use of Stem Cells as a Potential Treatment Method for Selected Neurodegenerative Diseases: Review. Cell Mol. Neurobiol..

[49] Rahman M.H., Akter R., Bhattacharya T., Abdel-Daim M.M., Alkahtani S., Arafah M.W., Al-Johani N.S., Alhoshani N.M., Alkeraishan N., Alhenaky A. (2020). Resveratrol and Neuroprotection: Impact and Its Therapeutic Potential in Alzheimer’s Disease. Front. Pharmacol..

[50] Zhang L.X., Li C.X., Kakar M.U., Khan M.S., Wu P.F., Amir R.M., Dai D.F., Naveed M., Li Q.Y., Saeed M. (2021). Resveratrol (RV): A pharmacological review and call for further research. Biomed. Pharmacother..

[51] Fan Y., Li Y., Huang S., Xu H., Li H., Liu B. (2020). Resveratrol-primed exosomes strongly promote the recovery of motor function in SCI rats by activating autophagy and inhibiting apoptosis via the PI3K signaling pathway. Neurosci. Lett..

[52] Zheng X., Sun K., Liu Y., Yin X., Zhu H., Yu F., Zhao W. (2023). Resveratrol-loaded macrophage exosomes alleviate multiple sclerosis through targeting microglia. J. Control Release.

[53] Singh G., Mehra A., Arora S., Gugulothu D., Vora L.K., Prasad R., Khatri D.K. (2024). Exosome-mediated delivery and regulation in neurological disease progression. Int. J. Biol. Macromol..

[54] Qi D., Hou X., Jin C., Chen X., Pan C., Fu H., Song L., Xue J. (2021). HNSC exosome-derived MIAT improves cognitive disorders in rats with vascular dementia via the miR-34b-5p/CALB1 axis. Am. J. Transl. Res..

[55] Hardie D.G. (2014). AMPK--sensing energy while talking to other signaling pathways. Cell Metab..

[56] Lin S.C., Hardie D.G. (2018). AMPK: Sensing Glucose as well as Cellular Energy Status. Cell Metab..

[57] Edlich F. (2018). BCL-2 proteins and apoptosis: Recent insights and unknowns. Biochem. Biophys. Res. Commun..

[58] Sakamoto K., Karelina K., Obrietan K. (2011). CREB: A multifaceted regulator of neuronal plasticity and protection. J. Neurochem..

[59] Ortega-Martinez S. (2015). A new perspective on the role of the CREB family of transcription factors in memory consolidation via adult hippocampal neurogenesis. Front. Mol. Neurosci..

[60] Choong C.J., Mochizuki H. (2023). Involvement of Mitochondria in Parkinson’s Disease. Int. J. Mol. Sci..

[61] Abu Shelbayeh O., Arroum T., Morris S., Busch K.B. (2023). PGC-1alpha Is a Master Regulator of Mitochondrial Lifecycle and ROS Stress Response. Antioxidants.

[62] Kang I., Chu C.T., Kaufman B.A. (2018). The mitochondrial transcription factor TFAM in neurodegeneration: Emerging evidence and mechanisms. FEBS Lett..

[63] Liu K., Li W., Xiao Y., Lei M., Zhang M., Min J. (2024). Molecular mechanism of specific DNA sequence recognition by NRF1. Nucleic Acids Res..

[64] Sun Y., Sukumaran P., Selvaraj S., Cilz N.I., Schaar A., Lei S., Singh B.B. (2018). TRPM2 Promotes Neurotoxin MPP(+)/MPTP-Induced Cell Death. Mol. Neurobiol..

[65] Liu M., Zuo S., Guo X., Peng J., Xing Y., Guo Y., Li C., Xing H. (2023). The Study of Overexpression of Peroxiredoxin-2 Reduces MPP(+)-Induced Toxicity in the Cell Model of Parkinson’s Disease. Neurochem. Res..

[66] Chhunchha B., Kubo E., Singh D.P. (2022). Obligatory Role of AMPK Activation and Antioxidant Defense Pathway in the Regulatory Effects of Metformin on Cellular Protection and Prevention of Lens Opacity. Cells.

[67] Xu W., Zhao T., Xiao H. (2020). The Implication of Oxidative Stress and AMPK-Nrf2 Antioxidative Signaling in Pneumonia Pathogenesis. Front. Endocrinol..

[68] Petsouki E., Cabrera S.N.S., Heiss E.H. (2022). AMPK and NRF2: Interactive players in the same team for cellular homeostasis?. Free Radic. Biol. Med..

[69] Chiang M.C., Nicol C.J.B., Lo S.S., Hung S.W., Wang C.J., Lin C.H. (2022). Resveratrol Mitigates Oxygen and Glucose Deprivation-Induced Inflammation, NLRP3 Inflammasome, and Oxidative Stress in 3D Neuronal Culture. Int. J. Mol. Sci..

[70] Forman H.J., Zhang H. (2021). Targeting oxidative stress in disease: Promise and limitations of antioxidant therapy. Nat. Rev. Drug Discov..

[71] Limanaqi F., Biagioni F., Gambardella S., Familiari P., Frati A., Fornai F. (2020). Promiscuous Roles of Autophagy and Proteasome in Neurodegenerative Proteinopathies. Int. J. Mol. Sci..

[72] McKinnon C., De Snoo M.L., Gondard E., Neudorfer C., Chau H., Ngana S.G., O’Hara D.M., Brotchie J.M., Koprich J.B., Lozano A.M. (2020). Early-onset impairment of the ubiquitin-proteasome system in dopaminergic neurons caused by alpha-synuclein. Acta Neuropathol. Commun..

[73] Yu J., Zhao Z., Li Y., Chen J., Huang N., Luo Y. (2024). Role of NLRP3 in Parkinson’s disease: Specific activation especially in dopaminergic neurons. Heliyon.

[74] Jewell S., Herath A.M., Gordon R. (2022). Inflammasome Activation in Parkinson’s Disease. J. Park. Dis..

[75] Brentnall M., Rodriguez-Menocal L., De Guevara R.L., Cepero E., Boise L.H. (2013). Caspase-9, caspase-3 and caspase-7 have distinct roles during intrinsic apoptosis. BMC Cell Biol..

[76] Chiang M.C., Nicol C.J.B., Yang Y.P., Chiang T., Yen C. (2025). Protective effects of resveratrol against PM(2.5)-induced damage in hNSCs and its mitigation of PM(2.5)-induced mitochondrial dysfunction in a 3D scaffold system. Neuroscience.

