# Supplementary material for: Resveratrol-Enhanced Human Neural Stem Cell-Derived Exosomes Mitigate MPP+-Induced Neurotoxicity Through Activation of AMPK and Nrf2 Pathways and Inhibition of the NLRP3 Inflammasome in SH-SY5Y Cells
Source: Life (Basel). 2025 Feb 13;15(2):294. doi: 10.3390/life15020294 (PMC11856727; doi:10.3390/life15020294)
Supplement: Supplementary file 1 [file life-15-00294-s001.zip › life-3422315-supplementary.pdf]

Supplementary File(s): Figures S1 and S2.

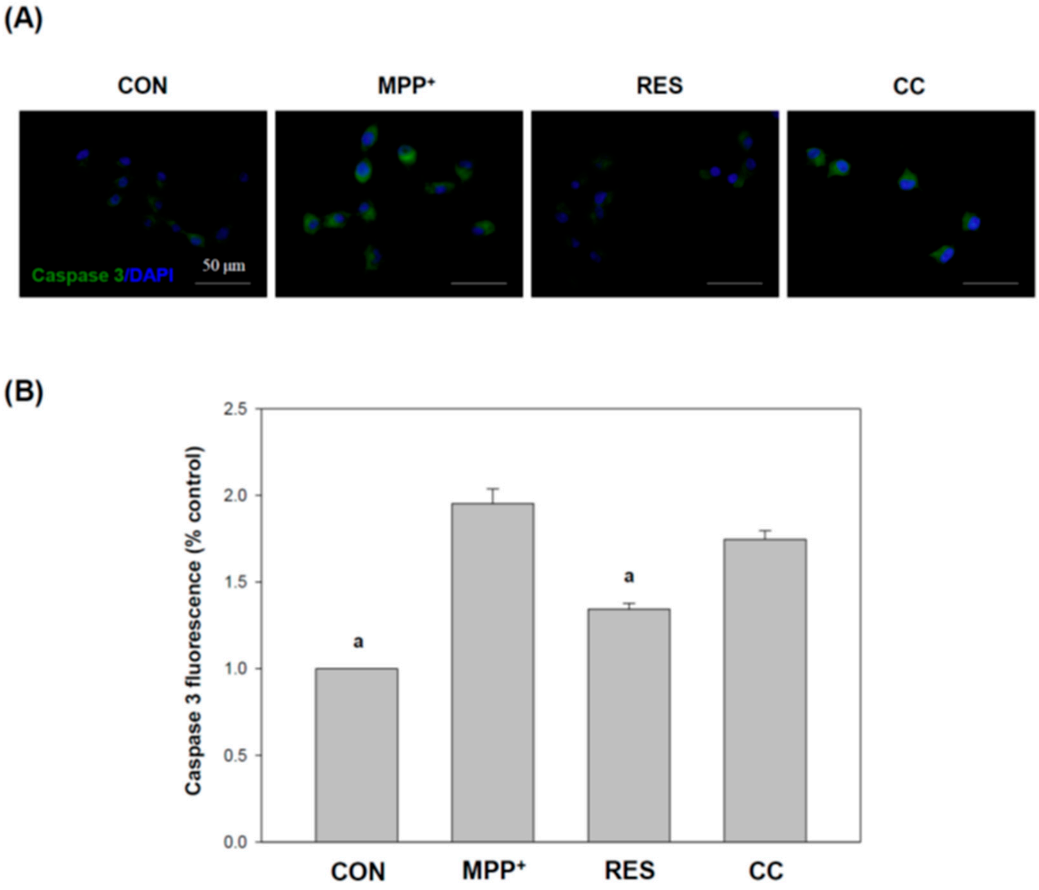

Figure. S1. Effects of hNSCs-Exos on Immunostaining Analysis of Caspase 3 Levels in SH-SY5Y Cells Exposed to MPP$^+$. Cells were treated following the protocol outlined in Figure 1 to assess caspase 3 protein levels and their cellular localization in SH-SY5Y cells exposed to MPP$^+$, with and without treatment. (A) Immunostaining: Antibodies against caspase 3 (green) were used to visualize its expression in SH-SY5Y cells. The nucleus was counterstained with DAPI (blue fluorescence). Representative images show distinct caspase 3 expression patterns across different treatment conditions, with a notable decrease in caspase 3 levels in cells treated with RES-hNSCs-Exos. The scale bar represents 50 µm. (B) Quantitative Analysis: The intensity of caspase 3 immunostaining was quantitatively analyzed, demonstrating significant differences in caspase 3 expression levels between treatment groups. Specifically, cells treated with RES-hNSCs-Exos showed a marked decrease in caspase 3 levels compared to cells treated with MPP$^+$ alone. The data represent mean ± SEM from three independent experiments. Statistical differences were determined as described in Figure 1.

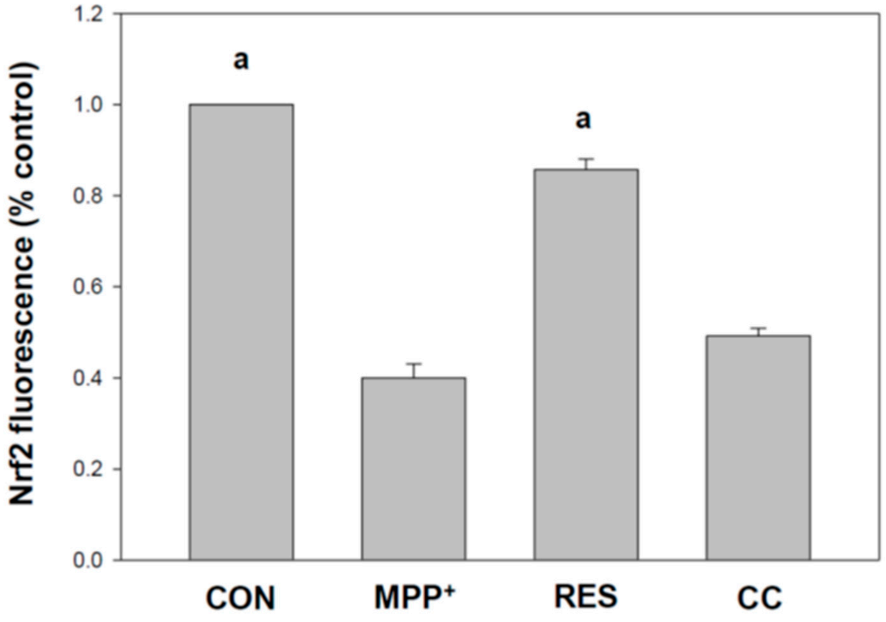

Figure. S2. Effects of hNSCs-Exos on Nrf2 Levels in SH-SY5Y Cells Exposed to MPP$^+$. Quantitative Analysis: The intensity of Nrf2 immunostaining was quantitatively analyzed, demonstrating significant differences in Nrf2 expression levels between treatment groups. Specifically, cells treated with RES-hNSCs-Exos showed a marked increase in Nrf2 levels compared to cells treated with MPP$^+$ alone. The data represent mean ± SEM from three independent experiments. Statistical differences were determined as described in Figure 1.
